# Synergistic Role of Water and Oxygen Leads to Degradation in Formamidinium-Based Halide Perovskites

**DOI:** 10.1021/jacs.3c05657

**Published:** 2023-11-02

**Authors:** Juanita Hidalgo, Waldemar Kaiser, Yu An, Ruipeng Li, Zion Oh, Andrés-Felipe Castro-Méndez, Diana K. LaFollette, Sanggyun Kim, Barry Lai, Joachim Breternitz, Susan Schorr, Carlo A. R. Perini, Edoardo Mosconi, Filippo De Angelis, Juan-Pablo Correa-Baena

**Affiliations:** † School of Materials Science and Engineering, Georgia Institute of Technology, Atlanta, Georgia 30332, United States; ‡ Computational Laboratory for Hybrid/Organic Photovoltaics (CLHYO), Istituto CNR di Scienze e Tecnologie Chimiche “Giulio Natta” (CNR-SCITEC), Perugia 06123, Italy; § National Synchrotron Light Source II, Brookhaven National Lab, Upton, New York 11973, United States; ∥ Advanced Photon Source, Argonne National Laboratory, Lemont, Illinois 60439, United States; ⊥ Department of Structure and Dynamics of Energy Materials, Helmholtz-Zentrum Berlin für Materialien und Energie, Hahn-Meitner-Platz 1, Berlin 14109, Germany; # Freie Universitaet Berlin, Institute of Geological Sciences, Malteser Str. 74-200, Berlin 12249, Germany; ¶ Department of Chemistry, Biology and Biotechnology, University of Perugia and UdR INSTM, Perugia 06123, Italy; ∇ Department of Natural Sciences & Mathematics, College of Sciences & Human Studies, Prince Mohammad Bin Fahd University, Dhahran 34754, Saudi Arabia; ○ SKKU Institute of Energy Science and Technology (SIEST), Sungkyunkwan University, Suwon 440-746, Korea

## Abstract

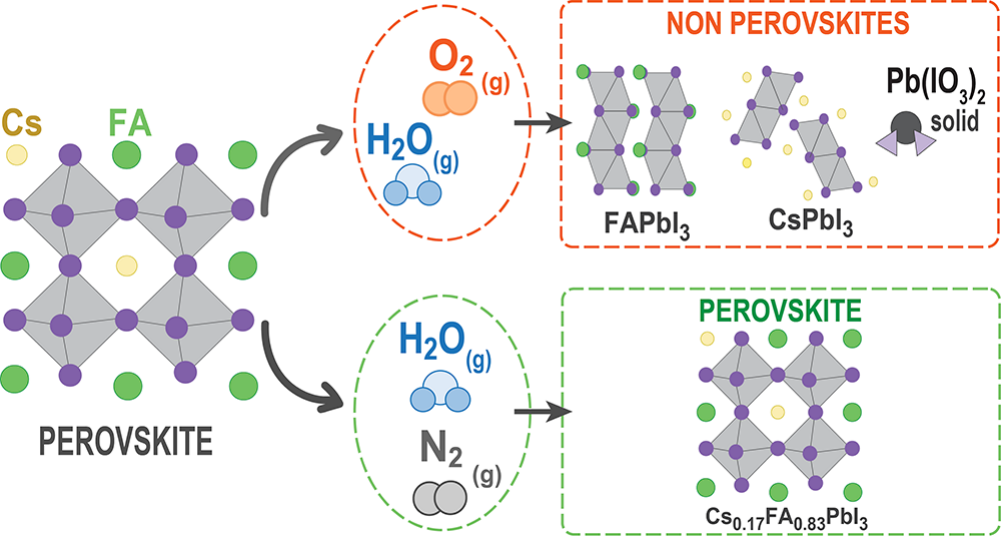

Mixed-cation metal halide perovskites have shown remarkable progress in photovoltaic applications with high power conversion efficiencies. However, to achieve large-scale deployment of this technology, efficiencies must be complemented by long-term durability. The latter is limited by external factors, such as exposure to humidity and air, which lead to the rapid degradation of the perovskite materials and devices. In this work, we study the mechanisms causing Cs and formamidinium (FA)-based halide perovskite phase transformations and stabilization during moisture and air exposure. We use *in situ* X-ray scattering, X-ray photoelectron spectroscopy, and first-principles calculations to study these chemical interactions and their effects on structure. We unravel a surface reaction pathway involving the dissolution of FAI by water and iodide oxidation by oxygen, driving the Cs/FA ratio into thermodynamically unstable regions, leading to undesirable phase transformations. This work demonstrates the interplay of bulk phase transformations with surface chemical reactions, providing a detailed understanding of the degradation mechanism and strategies for designing durable and efficient perovskite materials.

## 1 Introduction

Formamidinium [CH(NH_2_)_2_, FA] metal halide perovskites have emerged as promising materials for solar cell applications due to their exceptional light-harvesting properties, producing high power conversion efficiencies (PCEs) of over 26%.^1^ However, one of the most significant challenges is their poor stability, particularly in the presence of moisture (H_2_O) and oxygen (O_2_), which can trigger phase transformations to nonperovskite structures during solar cell fabrication and operation.^2^ To overcome this challenge, mixed ion perovskites, including Cs_*x*_FA_1–*x*_PbI_3_ (CsFA), have been shown to provide improved stability compared to their single-cation counterparts.^2−4^ However, CsFA perovskites also degrade when exposed to ambient air, where the perovskite structure transforms into nonperovskite phases such as one of the hexagonal FAPbI_3_ (2H) and orthorhombic δ-CsPbI_3_ (δCs) structures.^5,6^ Perovskite degradation due to water exposure^7−11^ and photo-oxidation under illumination in oxygen is widely recognized.^12−15^ Nonetheless, we lack a clear understanding of the mechanisms that lead to phase instabilities in these FA-rich perovskites due to water and oxygen interactions. Therefore, a fundamental understanding of the mechanisms causing perovskite phase transformations is crucial for developing durable and efficient metal halide perovskites for solar cell applications.

Herein, we investigate the origin of structural phase instability of FA-based perovskites upon exposure to H_2_O, with air (H_2_O/air) or nitrogen (H_2_O/N_2_) as carrier gases, using *in situ* grazing incidence wide-angle X-ray scattering (GIWAXS). We study the surface chemistry and propose a mechanism to explain phase transformations when the perovskites are exposed to H_2_O and O_2_ by using X-ray photoelectron spectroscopy (XPS) and density functional theory (DFT) calculations. We find that the degradation rate is considerably slower when the perovskite is exposed to H_2_O/N_2_ when compared to H_2_O/air. Our results show that a critical synergy between H_2_O and O_2_ in air is needed to accelerate the undesired phase transformations in perovskites. The H_2_O molecules dissolve FAI on the perovskite surface, leading to volatilization of the iodide and FA^+^ cations. The O_2_ may then interact with the exposed lead-iodide-rich surfaces, oxidizing iodide and forming the thermodynamically favored iodate species, IO_3_^–^, which bonds to surface Pb ions. Lead(II) iodate, Pb(IO_3_)_2_, can then form and segregate at the surface, leaving PbI_2_ vacancies behind and allowing H_2_O molecules to further infiltrate the crystal structure. The continuous removal of FAI causes a local imbalance between Cs^+^ and FA^+^, destabilizing the CsFA phase and favoring a transformation to the 2H and δCs phases. The degradation proceeds both in dark and light conditions, with light accelerating the formation of secondary phases. Finally, we show that a surface treatment using a hydrophobic top layer of phenethylammonium iodide (PEAI) effectively stabilizes the perovskite phase even in the presence of H_2_O/air. Solar cells made of CsFA-PEAI films exhibit stable PCEs even after exposure to H_2_O and air. Our study provides structural and atomistic insights into the phase instability in FA-based perovskites when exposed to humid air conditions and provides surface passivation strategies to stabilize the perovskite phase for highly stable and efficient solar cells.

## 2 Results

### 2.1 Structural Phase Transformations

**Figure 1:**
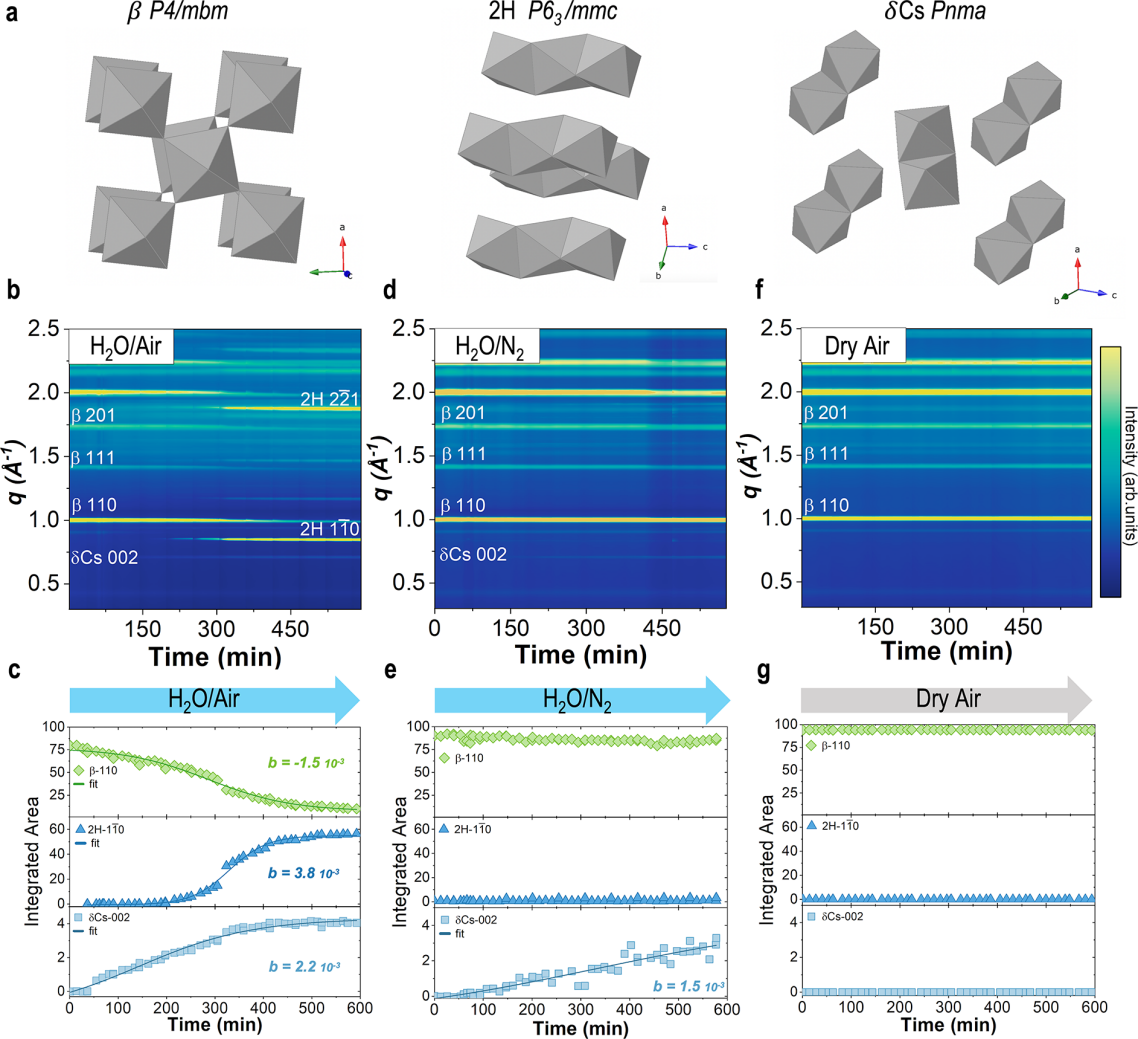
Humidity-induced structural phase transformations measured by *in situ* GIWAXS. The structural phases analyzed are (a) tetragonal β–perovskite of space group *P*4/*mbm* (left), 2H FAPbI_3_ hexagonal nonperovskite phase of space group *P*6_3_/*mmc* (center), and orthorhombic δ−CsPbI_3_ nonperovskite phase of space group *Pnma* (right). Phase transformations over time of CsFA β–perovskite exposed to (b) H_2_O/air, (d) H_2_O/N_2_, and (f) dry air, where (c, e, and g) are the corresponding integrated areas of the main scattering peaks of each phase.

To understand the structural phase transformations in FA-based perovskites, we used *in situ* GIWAXS, as shown in Figures 1 and S1, where we exposed CsFA films to a relative humidity of ∼100% and in dark conditions (lights off). Initially, we analyzed the structural phases without H_2_O or air exposure (Le Bail refinement, Figure S2, Table S1), resulting in a mixed-cation (Cs 17%—FA 83%) tetragonal perovskite phase (β) with a space group *P*4/*mbm*^5,16^ (Figure 1a). Figure 1b shows the *in situ* GIWAXS scattering patterns as a function of time exposed to H_2_O/air for 600 min. In H_2_O/air, the β phase (mixed-cation) transformed into two different phases, namely, the single-cation FAPbI_3_ hexagonal phase (2H)^5^ and CsPbI_3_ orthorhombic phase (δCs)^3^ (crystal structures shown in Figure 1a). The 2H and δCs are both nonperovskite phases given the lack of corner-sharing octahedra. Figure 1c shows the quantified peak evolution of the integrated area of the main scattering peak of the perovskite (β) and nonperovskite (2H, δCs) phases. We observe that δCs forms at around 50 min of exposure to H_2_O/air, while 2H forms after 200 min of exposure, but at a faster rate. A kinetic model was used to quantify the phase transformation rates, shown in Figures S3 and S4, where we fit a rate constant *b*. The negative *b* for the β-110 peak indicates the loss of the β-perovskite phase in H_2_O/air. The positive *b* for the 2H and δCs peaks reveals the appearance of the nonperovskite phases and a faster formation rate for 2H compared to δCs.

To isolate the effect of H_2_O from O_2_, we performed *in situ* GIWAXS experiments by exposing the CsFA films to H_2_O/N_2_, as shown in Figure 1d. It is important to study the role of H_2_O alone, considering that O_2_ has been key in the photo-oxidation of methylammonium lead iodide (MAPbI_3_) under illumination.^17−19^ Remarkably, when CsFA is exposed to H_2_O/N_2_, the β phase does not change, and a 2H phase does not appear. This is in contrast with the samples exposed to H_2_O/air, which suffered phase transformations after the same exposure time. The peak evolution in Figure 1e shows a minor decrease in the β-perovskite integrated area from 92 to 85 with no formation of the 2H phase. However, the δCs phase still forms with a rate constant *b* of 1.5, compared to 2.2 in H_2_O/air (Figure S4), confirming a slower δCs phase formation in H_2_O/N_2_.

To isolate the role of oxygen (from air) from that of the water molecule, we exposed the CsFA films to dry air only. Interestingly, from *in situ* GIWAXS (Figure 1f) and the time evolution of the main peaks (Figure 1g), the phase transformations previously seen do not occur. This reveals that the amount of photo-oxidation induced by the X-rays alone is not sufficient to induce the formation of secondary nonperovskite phases. The effects induced by humidity or dry air exposure are independent of the substrate we use (Figure S5). These result show that the CsFA phase instability is not just due to humidity exposure but is accelerated by O_2_. These results are further corroborated by additional analyses of the *in situ* GIWAXS experiments (Figures S5 and S6). It is worth noting that X-ray beam-induced damage due to prolonged exposure during *in situ* experiments can provide artifacts to measurements, as it has been shown in a wide variety of materials.^20,21^ Thus, to deconvolute potential effects on the structure caused by X-rays, we measured GIWAXS in a separate isolated location of the same film (without X-ray exposure) to make sure the peak intensity did not change due to beam damage. The second isolated location was measured in parallel and with lower beam exposure, given that the data were taken with longer delays between measurements. The data for the isolated location is summarized in Figure S7 and shows a very similar trend to the consecutive measurements shown in Figure 1.

To study the structural changes at the surface of CsFA films exposed to H_2_O/air, we analyzed the *in situ* GIWAXS with an incident angle below the critical angle (Figure S8). We observed the same transformation from perovskite into nonperovskites on the surface as in the bulk (Figure 1). Furthermore, analyzing the formation of the nonperovskite phases 2H and δCs, we calculated a larger rate constant *b* at the surface (Figure S8), evidence of a faster transformation into nonperovskites. In addition, areas with more charging were observed by scanning electron microscopy (Figure S9), suggesting the formation of new phases. UV–vis spectroscopy shows a larger band gap for the CsFA film after H_2_O/air exposure, which may be due to the absorption from the 2H phase (Figure S10).^22,23^ The emission observed from both photoluminescence (PL) and carrier lifetime from time-resolved PL (TRPL) is reduced, as expected with the conversion to nonperovskite phases (Figure S10).

### 2.2 Surface Chemistry

**Figure 2:**
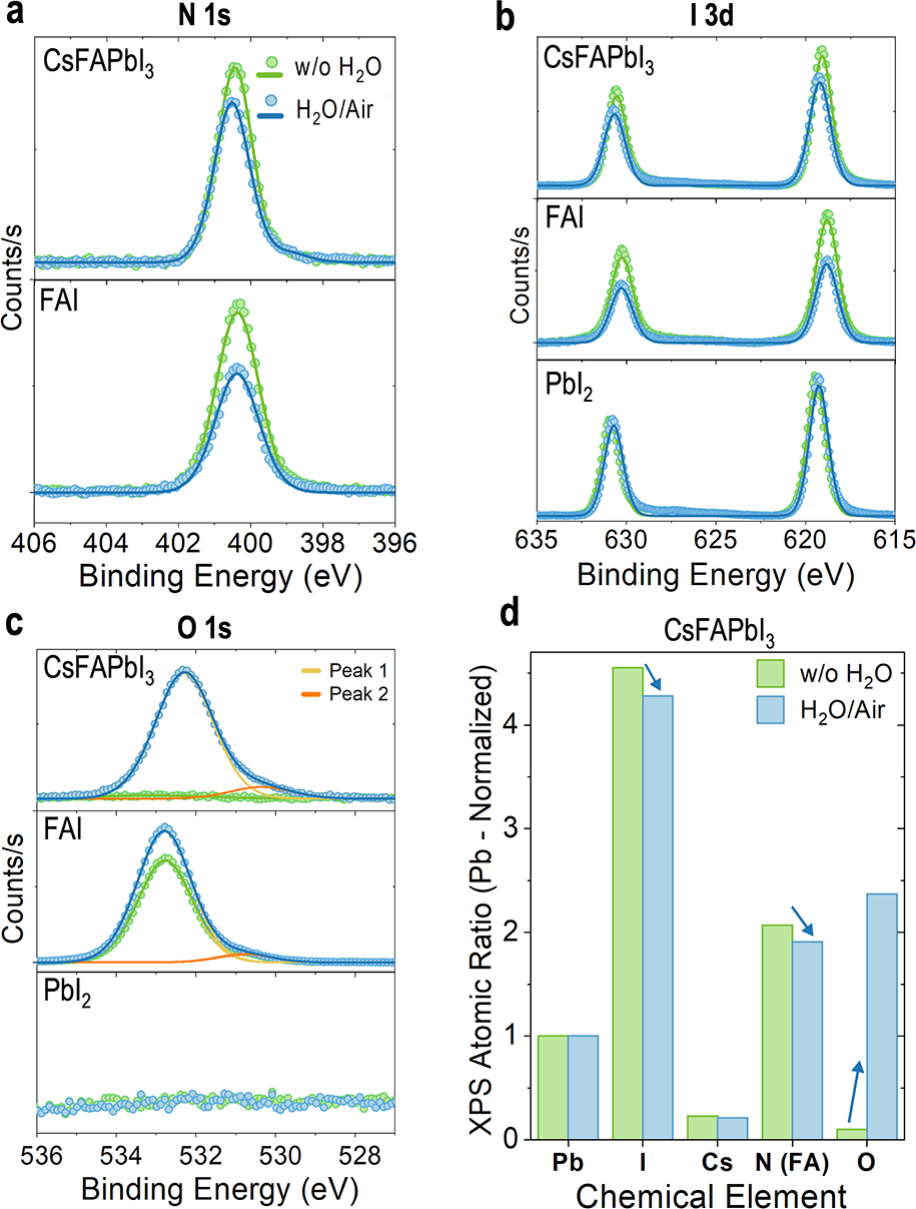
XPS spectra of the peaks: (a) N 1s, (b) I 3d, and (c) O 1s of CsFA perovskite, FAI, and PbI_2_ films without (w/o) and after H_2_O/air exposure. For the CsFA perovskite films, panel (d) shows the atomic ratio of iodine(I), cesium (Cs), nitrogen (N, FA), or oxygen (O) normalized to lead (Pb), for pristine films and after H_2_O/air exposure. Full peak deconvolution and details for the CsFA films can be found in Figures S10 and S11 and Tables S2 and S3, for FAI films in Figure S12, Tables S4 and S5, and for **PbI**_**2**_ films in Figure S13 and Tables S6 and S7. CsFA perovskite films w/o H_2_O exposure (green) were fabricated in a nitrogen glovebox and exposed to a room atmosphere while mounting the XPS measurement. CsFA films after H_2_O/air exposure (blue) were fabricated in a nitrogen glovebox, exposed to humidity, and then measured.

To investigate the chemical changes at the surface, we performed XPS of the CsFA films without exposure and after H_2_O/air exposure (Figure 2).^21^ After exposure to H_2_O/air, the N 1s peak (Figure 2a, CsFA) decreases by 5.6% in atomic content (Tables S2 and S3), evidence of the FA^+^ volatilization, as the N 1s peak corresponds to the C=N bond of the FA.^24^ The I 3d peak from iodine decreases by 11.4% in atomic content after exposure to H_2_O/air (Figure 2b, CsFA), suggesting the loss of FAI at the surface and the additional loss of iodine from elsewhere in the structure.^19,25,26^

The exposure of CsFA films to H_2_O/air influences the oxygen signal. An increase in the intensity of the peak signal of the O 1s is observed after exposure (Figure 2c), corresponding to an 18% atomic content (Table S3). The O 1s peak (peak 1) is centered at 532.3 eV, suggesting the existence of adsorbed O_2_ molecules.^27^ Peak 1 may be attributed to the formation of hydroxides (OH^–^) or carbonates (CO_3_^2-^) expected from the exposure to H_2_O/air.^28^ The C 1s peaks also suggest carbon–oxygen complexes such as carbonates after H_2_O/air exposure (Figure S11).^27^ The peak assigned to C–C or C–H bonds increases after H_2_O/air exposure, possibly from adventitious carbon (Figure S12). Figure 2c also shows a small peak 2, centered at 530.4 eV, suggesting the formation of Pb-oxides.^27^Figure 2d summarizes the changes in the atomic ratio of iodine, cesium, nitrogen, and oxygen compared to Pb on CsFA films after H_2_O/air exposure, showing the volatilization of nitrogen and iodine, and an increase in oxygen content.

To further understand the chemical surface species after H_2_O/air exposure, we deposited FAI and PbI_2_ thin films and characterized their surface chemistries by XPS (Figure 2a-c FAI, PbI_2_, and Figures S13 and S14). After H_2_O/air exposure, the FAI films show a decrease in the atomic content from N 1s and I 3d, confirming the volatilization of FAI (Figure 2a,b, FAI, and Tables S4 and S5). An increase in the intensity of the O 1s peak is observed for the FAI films (Figure 2c, FAI). The FAI films without H_2_O exposure show an O 1s peak centered at 532.8 eV in Figure 2c (FAI), suggesting that atmospheric H_2_O and O_2_ molecules are adsorbed to the FAI films when the samples are mounted in the XPS, confirming the high hydrophilicity of FAI. The deconvolution of the O 1s peak shows a second peak (peak 2) centered at 530.8 eV, indicating that this peak may result from FA exposure to ambient conditions. Therefore, the O 1s peak 2 in Figure 2c (CsFA) can be attributed to either Pb-oxides and to I- or FA-based oxides. In contrast, the PbI_2_ films before and after H_2_O/air exposure do not show changes in Pb 4f (Figure S14) or I 3d (Figure 2b, PbI_2_), and there is no oxygen from the O 1s spectrum (Figure 2c, PbI_2_ and Tables S6 and S7). We studied the chemistry changes in the bulk by X-ray fluorescence (XRF) elemental mapping, which also shows the loss of iodine after H_2_O/air exposure (Figure S15), agreeing with the XPS results (Figure 2b). Fourier transform infrared spectra (FTIR) showed no oxygen in the vibrational modes of CsFA powders and films (Figures S16 and S17, Table S8). Thus, we are confident that the H_2_O/air-induced CsFA phase transformations begin at the surface through the interaction of oxygen with the perovskites.

### 2.3 Chemical Reaction Mechanism

Previous studies have suggested adverse effects of H_2_O on lead-iodide perovskite surfaces leading to the degradation of the material.^6,17,29,30^ Molecular dynamics simulations of MAPbI_3_/H_2_O interfaces have shown the fast dissolution of MAI-terminated surfaces, as H_2_O molecules break the bond between surface I^–^ and underlying Pb^2+^ ion resulting in the removal of I^–^, accompanied by the desorption of the MA^+^.^31,32^ Despite FA being less polar than MA,^33^ the same mechanism is expected to occur on FAI-terminated surfaces. Previous results showed the dissolution of FAPbI_3_ with water vapor,^34^ explaining the release of I^–^ and FA^+^ from the CsFA perovskite surface^31^ and within the FAI films (Figure 2a,b,d) after exposure to H_2_O/air.^32^ The removal of CsI or FAI leads to a PbI_2_-terminated surface that is more resistant to degradation by water alone.^31^

**Figure 3:**
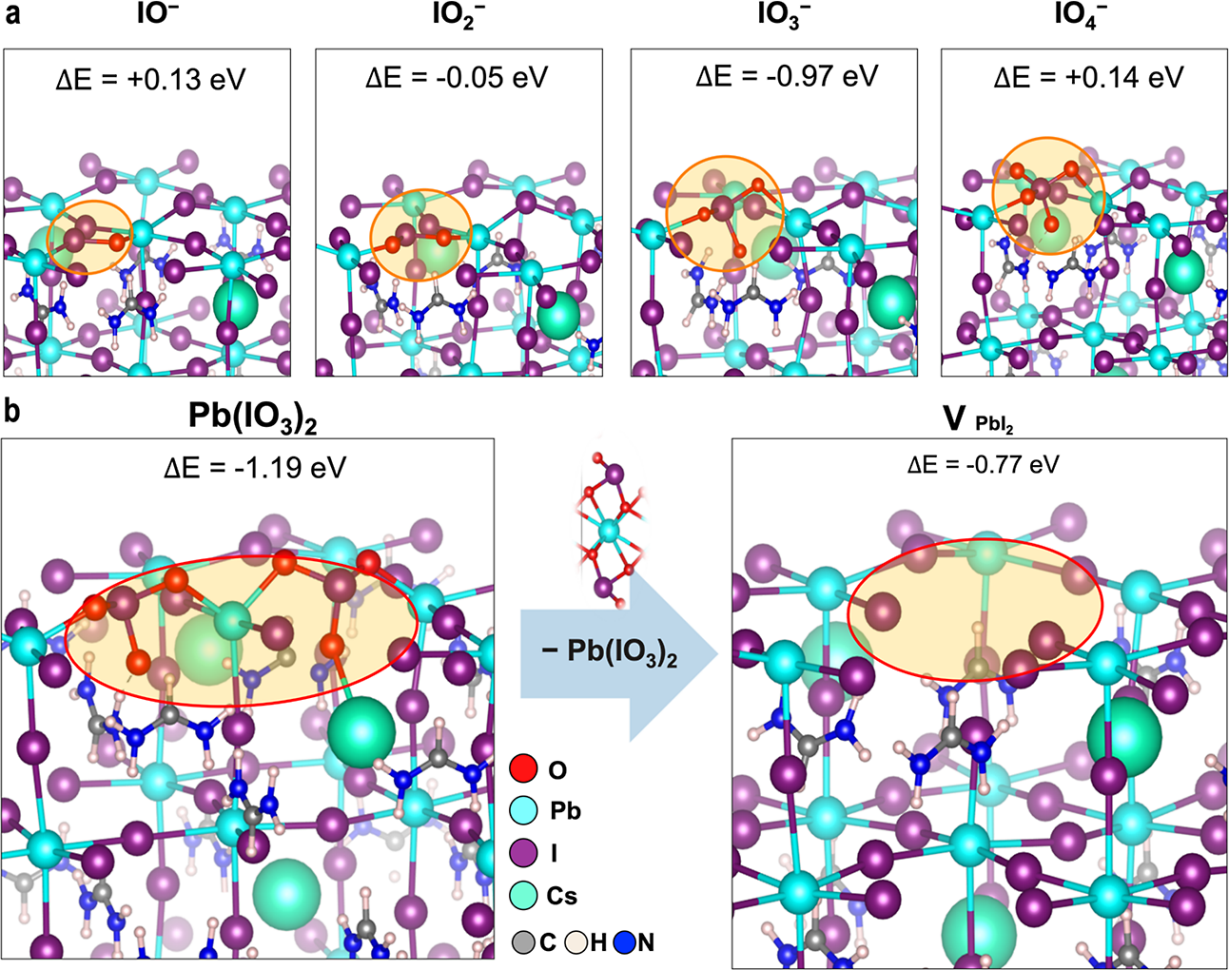
DFT calculations of iodide oxidation and superoxide formation on the perovskite surface. Panel (a) visualizes the formation of IO_*n*_^–^ species upon oxidation of surface iodide ions [Rx. 1]; from left to right: hypoiodite, IO^–^; iodite, IO_2_^–^; iodate, IO_3_^–^; and periodate, IO_4_^–^. All oxidized species are explicitly highlighted, and reaction energies are given (see the computational methods for details). (b) Reaction mechanism [Rx. 2 and 3] of (left) lead(II) iodate, Pb(IO_3_)_2_, formation by consumption of oxygen molecules and (right) removal of Pb(IO_3_)_2_ resulting in a surface vacancy V_PbI2_. The following color code is used for the atomic representations: purple, I; cyan, Pb; blue, N; green, Cs; gray, C; white, H; and red, O.

To understand the phase transformation mechanisms, we performed DFT calculations on the interactions of oxygen molecules with the PbI_2_-terminated CsFA perovskite surface (Figure S18). Our calculations suggest that the O_2_ adsorption is favored at the PbI_2_-terminated surface (*E*_ads_ = −0.03 to −0.22 eV, Table S9) compared to a CsFAI-terminated surface (*E*_ads_ = 0.06 to 0.36 eV, Table S10), while H_2_O may further support O_2_ adsorption. Note that the PbI_2_-terminated surface is made of undercoordinated Pb^2+^, which makes it more hydrophilic than the PbI_2_ crystal phase (Figure 2c) made of fully coordinated Pb^2+^.^35^ The direct formation of PbO units from the adsorption of O_2_ appears thermodynamically unfavorable, with reaction energies of +1.09 eV (Figure S19). Therefore, we consider the oxidation of iodide ions on the surface by O_2_ as the starting point, as modeled by reaction 1. Here, surface iodide changes its oxidation state from −1 to positive values (−1 + 2 × *n*) while oxygen atoms are in their stable −2 oxidation state. We observe that, as previously suggested,^36^ oxygen breaks the Pb–I bonds at the surface, forming a Pb–O–I_ox_–I^–^ bond network, where I_ox_ is the oxidized iodine and I^–^ is a surface iodide in its negative charge state as shown in Figure 3a.
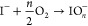
1

Our calculations show positive reaction energies for the formation of hypoiodite (IO^–^) and periodate (IO_4_^–^) of 0.13 and 0.14 eV, respectively, while iodite (IO_2_^–^) is slightly favored by −0.05 eV. Notably, iodate (IO_3_^–^) with iodide in its +5-oxidation state is strongly thermodynamically favored with a formation energy of −0.97 eV, indicating an irreversible surface transformation. From IO_3_^–^ in reaction 2, we consider the formation of lead(II) iodate, Pb(IO_3_)_2_, at the surface (indicated by an asterisk) by oxidizing two adjacent iodide ions sharing bonds with the same Pb surface ion. The formation energy of surface Pb(IO_3_)_2_ is Δ*E* = −1.19 eV (Figure 3b), which suggests a thermodynamically favorable replacement of a surface PbI_2_ unit. Finally, in reaction 3, we compute the removal of surface Pb(IO_3_)_2_ leaving a PbI_2_ vacancy (Figure 3b).^37^ We predict an energy of −0.77 eV for reaction 3, significantly more favorable than the direct removal of a PbI_2_ unit from a nonoxidized surface (Δ*E* = 0.07 eV, Figure S20).
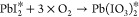
2
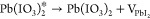
3

We note that the interaction of O_2_ with water molecules lowers the π* orbitals of O_2_ acting as accepting orbitals in oxidation reactions (Table S10), while hydration of perovskite surfaces raises the energies of the iodide-based valence band edge (Figure S21), which results in easier oxidation of surface iodide. These results suggest that O_2_ can modify PbI_2_-terminated surfaces by oxidizing iodide to iodate species and creating PbI_2_ vacancies. These vacancies may allow H_2_O molecules to enter the structure and dissolve the next FAI layers in an iterative process.

Our experimental and theoretical analyses suggest that first the H_2_O/air atmosphere dissolves the surface of CsFA perovskite and causes a loss in FA at the surface, likely increasing the relative amount of Cs relative to FA. Previous studies have shown that the CsFA perovskite phase becomes thermodynamically unstable when exceeding a Cs fraction of 0.2 (FA below 0.8).^3,4^ We note that the studied composition of Cs_0.17_FA_0.83_ is on the upper limit of Cs-molar content to form a single-phase perovskite.^4,5^ The loss of FAI may easily shift the composition into the thermodynamically unstable phase by increasing the ratio between Cs and FA, favoring the segregation into nonperovskite phases. Iodide vacancies created by H_2_O may further accelerate the phase transitions.^10^ Light can speed up these reactions, but degradation can also happen in the dark. This is confirmed both by the trend in the XPS spectra, where exposure to H_2_O/air is performed in the dark, and by GIWAXS measurements performed on the same sample but in different spots and using different measurement frequencies (different X-ray doses), Figure S7. Previous studies have highlighted the role of superoxide on perovskite degradation under light irradiation.^13,19,25,38^ Both room light and X-ray exposure can photoexcite charge carriers during the *in situ* GIWAXS measurements, but they only lead to a negligible amount of free charge carriers and superoxide formation (discussion in S2.5, Figures S22–S26).

**Figure 4:**
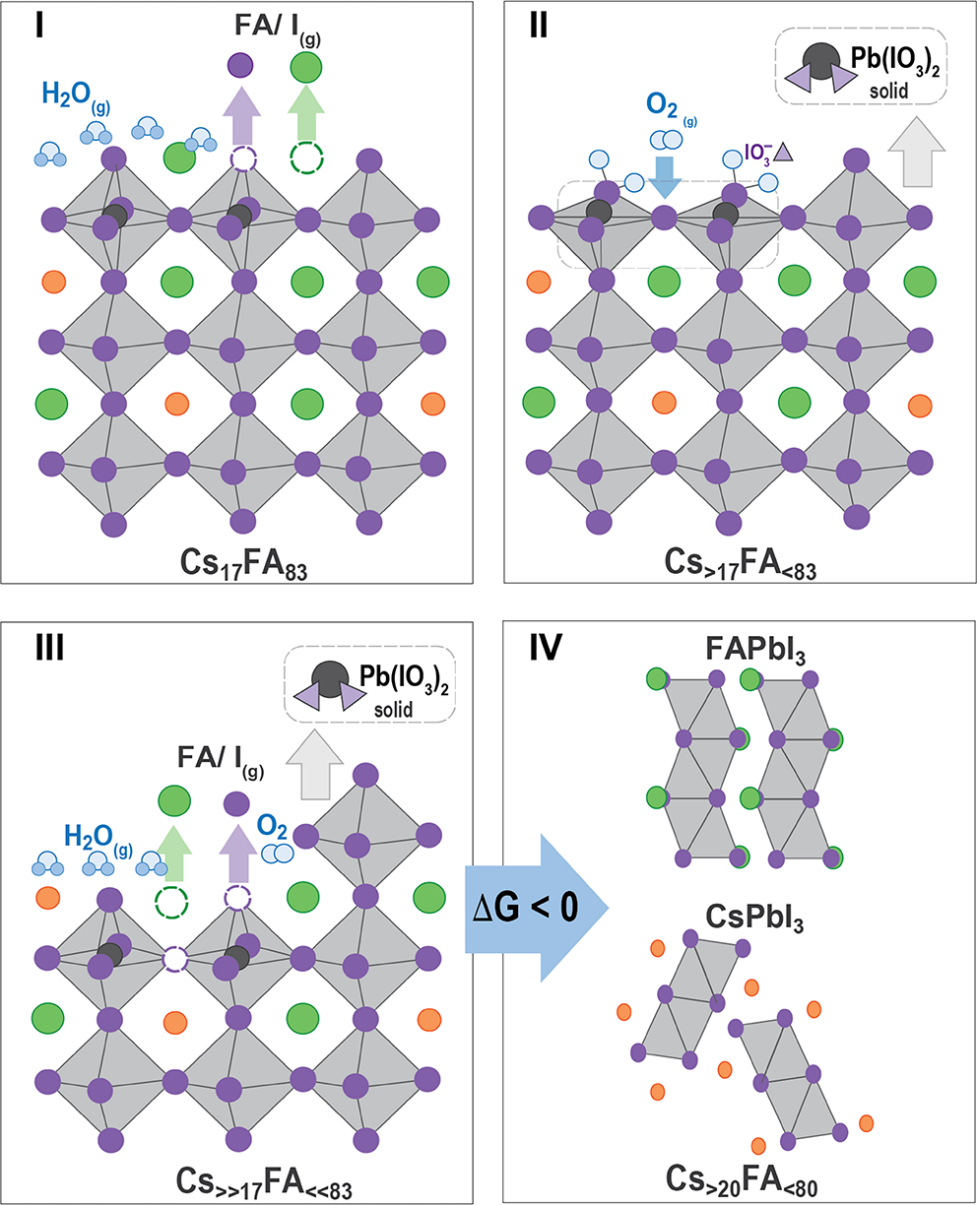
Proposed surface and bulk mechanism for CsFA perovskites exposed to H_2_O/air. (I) H_2_O is adsorbed on the CsFA surface, promoting the loss of FAI (g). Surface vacancies are created, leading to preferential oxygen binding sites, favoring the oxidation of iodide and energetically favorable formation of Pb(IO_3_)_2_ (II), which will create a PbI_2_ vacancy (III). Surface vacancies and the loss of FAI will lead to faster phase segregation and phase transformations from mixed-cation perovskite into single-cation nonperovskite phases (IV).

A proposed mechanism for the role of chemistry in phase transformations during exposure to H_2_O/air is shown in Figure 4. First (I), water molecules dissolve FAI at FAI-rich perovskite surfaces, creating PbI_2_-rich surface regions. Second (II), the O_2_ molecules are adsorbed and oxidize surface iodide, resulting in iodate species at the surface. Third (III), lead iodate can subsequently segregate at the surface, leaving PbI_2_ vacancies, which act as hotspots for further FAI dissolution by H_2_O. Fourth (IV), the loss of FA changes the Cs/FA molar ratio beyond the thermodynamically stable region, forming single-cation nonperovskite phases. We emphasize that the structural phase transformations in H_2_O/air are initiated at the surfaces. Thus, chemical surface treatments, such as reducing undercoordinated Pb ions at the surface, should reduce the interaction with H_2_O and O_2_, likely improving phase stabilization.

### 2.4 Stabilizing the Perovskite Phase

**Figure 5:**
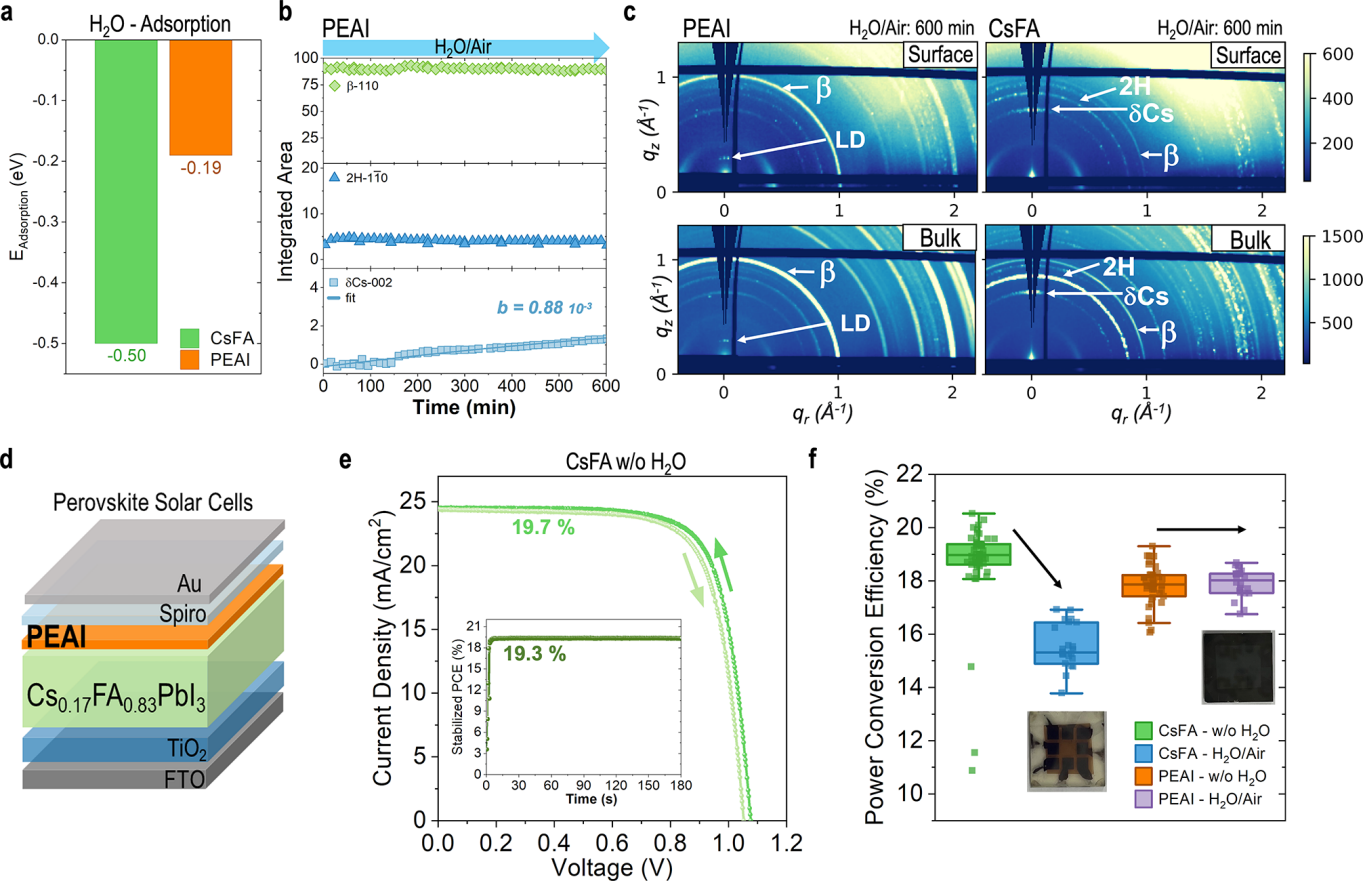
The PEAI capping layer is used to stabilize the perovskite in H_2_O/air. Panel (a) shows H_2_O adsorption energy on a CsFA surface and CsFA-PEAI surface calculated by DFT. From GIWAXS measurements in Figure S27, (b) is the integrated area of the main scattering peak of each phase for films exposed to H_2_O/air. (c) Shows the 2D GIWAXS patterns from the surface and bulk measurements after 600 min of exposure to H_2_O/air for the (left) PEAI-treated CsFA films and (right) untreated CsFA. A PEAI-treated CsFA perovskite layer in a solar cell with (d) n-i-p architecture. Panel (e) shows the current density–voltage curve and stabilized PCE of a high-efficiency device, and (f) shows the statistics of the PCE in box plots for the CsFA-untreated and PEAI-treated, w/o and with H_2_O exposure, under 1 sun illumination. The inset pictures show the device after H_2_O/air exposure before depositing the Spiro-OMeTAD and Au layers.

To understand the role of surface blockers, capping layers, or surface passivators on phase transformations at the interface,^39,40^ we evaluated the effect of PEAI spin-coated on a perovskite film (PEAI-treated).^41,42^ DFT calculations show a substantial reduction in H_2_O adsorption energies at the aromatic PEA^+^ (Figure 5a), resulting in hydrophobic protection that can reduce the degradation of CsFA perovskite surfaces (Figure S27). We analyzed the structural phase transformations by *in situ* GIWAXS in H_2_O/air (Figures S28–S30). Figure 5b shows the peak evolution of the main perovskite and nonperovskite phases (Figure 1a) as a function of exposure time to H_2_O/air. The main 110 β-perovskite peak does not change in H_2_O/air after 600 min of exposure, suggesting that the PEAI layer prevents H_2_O and O_2_ interactions at the surface compared to the untreated films in H_2_O/air (Figure 1b,c). Films treated with PEAI show a slight presence of 2H phase (Figure 5b), which decreases as a function of time (Figure S30). The δCs phase forms when exposed to H_2_O/air but three times slower than the untreated films (Figure 1c) when comparing the rate constant *b* (Figures S2 and S30). Figure 5c shows the 2D GIWAXS after final exposure to H_2_O/air. For the PEAI-treated films, the β-perovskite 110 Debye–Scherrer ring has a high intensity at the bulk and surface. We also observe rings that we assigned to low-dimensional (LD) PEA phases, as expected.^43^ The pristine CsFA films in Figure 5c show that the primary phases are 2H and δCs.

We evaluated the effect of the PEAI treatment on the performance and stability of solar cells of the n-i-p architecture (Figure 5d). CsFA-untreated devices showed an initial PCE of 19.7% (Figure 5e). The average PCE for the CsFA-untreated solar cells dropped 3.7% after H_2_O/air exposure (CsFA-H_2_O/air), while the average PCE for the PEAI-treated devices did not decrease (PEAI-H_2_O/air) (Figure 5f). A change in film color was visible after the pristine films were exposed to H_2_O/air (images in Figure 5f). The drop of PCE in CsFA-H_2_O/air is mainly due to the decrease in short circuit current density (Figure S31). We attribute this decrease to the formation of wide band gap nonperovskite phases such as 2H and δCs, which absorb less photons and thus transport fewer charge carriers in the solar cell. From correlative XRF and X-ray beam-induced current (XBIC) maps in Figure S32, we observe the formation of Cs-rich clusters that could correspond to δ-CsPbI_3_. The δCs clusters in XRF are correlated with reduced XBIC currents, in agreement with other studies.^44^ We assessed the long-term stability under operating conditions of solar cells^14,38^ under one sun illumination in dry air (Figures S33 and S34). Our results show an 85% decrease after 13 h in the untreated film solar cells. The PEAI-treated solar cells decreased only 30% from their initial PCE after 25 h, showing a slower degradation. This is in agreement with previous studies that highlighted the relevance of photo-oxidation of perovskite under 1 sun illumination and O_2_ exposure and further suggests increased robustness to the exposure to H_2_O/air of PEAI-treated surfaces (Figure S32).^13,19,25,38^ In contrast, the solar cells exposed to dry nitrogen showed little to no degradation (Figure S33), suggesting that oxygen in air is key to the degradation of the solar cells.

## 3 Conclusions

Exposing mixed-cation CsFA perovskites to H_2_O/air leads to undesired structural phase transformations, unlike the slower degradation observed in H_2_O/N_2_ and little to no degradation in dry air and the dark. When exposed to H_2_O/N_2_, the CsFA perovskite degrades more slowly by dissolving FAI molecules from the surface. However, in the presence of both H_2_O and air, after the volatilization of the FAI molecules, the O_2_ from air oxidizes surface iodide ions, forming lead(II) iodate. This further causes the formation of PbI_2_ vacancies, which act as hotspots for H_2_O to enter the structure and that lead to the volatilization of additional FAI molecules. This results in the loss of FAI molecules in an iterative process. This alteration in the local composition leads the Cs/FA ratio beyond the energetically stable region, producing a thermodynamic force that drives the phase transformation from perovskite to nonperovskite phases. This phase transformation process starts at the surface, where H_2_O and O_2_ react with surface ions. These insights into the surface chemistry and reaction mechanisms provide a foundation for designing durable and efficient solar cell materials. As a demonstration, a hydrophobic PEAI layer can be used to protect the surface from water and oxygen molecules, which prevents structural phase transformations and helps to preserve the solar cell performance.

## Supporting Information Available

All experimental methods and additional supporting results, *in situ* relative humidity GIWAXS measurements, Le Bail analysis of powder and thin-film diffraction data, lattice parameters from Le Bail analysis, integrated circular average from in situ relative humidity for different substrates and exposure conditions, GIWAXS measurement in a separate isolated location measured in parallel and with lower beam exposure, structural phase analysis at the surface, morphology from SEM in CsFA thin films w/o H_2_O exposure and after exposed ex-situ to H_2_O/air, optical properties of the CsFA exposed ex-situ to H_2_O/air, XPS spectra of CsFA films w/o exposure to H_2_O and exposed ex-situ to H_2_O/air, XPS convoluted peaks’ details and atomic percent for CsFA and FAI and PbI_2_ films w/o exposure to H_2_O and with exposure to H_2_O/air, XPS of deposited FAI and PbI_2_ thin films, XRF maps of the molar ratios between Cs, Pb, and I in CsFA thin films, FTIR spectra of CsFA thin films and powders, FTIR peaks and assignment for powders and thin films, surface models of CsFA with PbI_2_ and CsFAI termination, adsorption energy of O_2_ on PbI_2_- and CsFAI-terminated perovskite surfaces, DFT calculation of PbO formation at a defective PbI_2_-terminated surface and of PbI_2_ vacancy formation, energy of π* orbitals of molecular oxygen, DOS of the perovskite slab for the pristine and hydrated surface, top and side view of the FAI-terminated perovskite and the PbI_2_-terminated perovskite surface (DFT), DOS from spin-polarized DFT calculations for the pristine, hydrated, and defective surfaces after O_2_ adsorption, superoxide formation energies, H_2_O adsorption on the PEAI-terminated surface of a two-dimensional PEA_2_PbI_4_ perovskite and PbI_2_-terminated surface of the considered CsFA perovskite, *in situ* GIWAXS in H_2_O/air of PEAI-treated samples, simulated diffraction files for LD PEA cation Ruddlesden Popper phases, current-voltage curves for solar cells for the untreated CsFA perovskites and for the PEAI-treated without H_2_O exposure and after ex-situ H_2_O/air exposure, XRF with correlative XBIC done on complete CsFA solar cell devices, and long-term stability of the solar cells of CsFA without and with a top layer of PEAI (PDF)

## References

[ref1] Park J.; Kim J.; Yun H.-S.; Paik M. J.; Noh E.; Mun H. J.; Kim M. G.; Shin T. J.; Seok S. I. Controlled Growth of Perovskite Layers with Volatile Alkylammonium Chlorides. Nature 2023, 616, 724–730. 10.1038/s41586-023-05825-y. 36796426

[ref2] An Y.; Hidalgo J.; Perini C. A. R.; Castro-Méndez A. F.; Vagott J. N.; Bairley K.; Wang S.; Li X.; Correa-Baena J. P. Structural Stability of Formamidinium- And Cesium-Based Halide Perovskites. ACS Energy Lett. 2021, 6 (5), 1942–1969. 10.1021/acsenergylett.1c00354.

[ref3] Charles B.; Weller M. T.; Rieger S.; Hatcher L. E.; Henry P. F.; Feldmann J.; Wolverson D.; Wilson C. C. Phase Behavior and Substitution Limit of Mixed Cesium-Formamidinium Lead Triiodide Perovskites. Chem. Mater. 2020, 32 (6), 2282–2291. 10.1021/acs.chemmater.9b04032.

[ref4] Li Z.; Yang M.; Park J. S.; Wei S. H.; Berry J. J.; Zhu K. Stabilizing Perovskite Structures by Tuning Tolerance Factor: Formation of Formamidinium and Cesium Lead Iodide Solid-State Alloys. Chem. Mater. 2016, 28 (1), 284–292. 10.1021/acs.chemmater.5b04107.

[ref5] Weber O. J.; Ghosh D.; Gaines S.; Henry P. F.; Walker A. B.; Islam M. S.; Weller M. T. Phase Behavior and Polymorphism of Formamidinium Lead Iodide. Chem. Mater. 2018, 30 (11), 3768–3778. 10.1021/acs.chemmater.8b00862.

[ref6] Ho K.; Wei M.; Sargent E. H.; Walker G. C. Grain Transformation and Degradation Mechanism of Formamidinium and Cesium Lead Iodide Perovskite under Humidity and Light. ACS Energy Lett. 2021, 6 (3), 934–940. 10.1021/acsenergylett.0c02247.

[ref7] Zheng C.; Rubel O. Unraveling the Water Degradation Mechanism of CH3NH3PbI3. J. Phys. Chem. C 2019, 123 (32), 19385–19394. 10.1021/acs.jpcc.9b05516.

[ref8] Kye Y. H.; Yu C. J.; Jong U. G.; Chen Y.; Walsh A. Critical Role of Water in Defect Aggregation and Chemical Degradation of Perovskite Solar Cells. J. Phys. Chem. Lett. 2018, 9 (9), 2196–2201. 10.1021/acs.jpclett.8b00406. 29642701

[ref9] Shirayama M.; Kato M.; Miyadera T.; Sugita T.; Fujiseki T.; Hara S.; Kadowaki H.; Murata D.; Chikamatsu M.; Fujiwara H. Degradation Mechanism of CH3NH3PbI3 Perovskite Materials upon Exposure to Humid Air. J. Appl. Phys. 2016, 119 (11), 115501 10.1063/1.4943638.

[ref10] Lin J.; Lai M.; Dou L.; Kley C. S.; Chen H.; Peng F.; Sun J.; Lu D.; Hawks S. A.; Xie C.; Cui F.; Alivisatos A. P.; Limmer D. T.; Yang P. Thermochromic Halide Perovskite Solar Cells. Nat. Mater. 2018, 17 (3), 261–267. 10.1038/s41563-017-0006-0. 29358645

[ref11] Kaiser W.; Ricciarelli D.; Mosconi E.; Alothman A. A.; Ambrosio F.; De Angelis F. Stability of Tin- versus Lead-Halide Perovskites: Ab Initio Molecular Dynamics Simulations of Perovskite/Water Interfaces. J. Phys. Chem. Lett. 2022, 13 (10), 2321–2329. 10.1021/acs.jpclett.2c00273. 35245058 PMC8935372

[ref12] Zhang L.; Sit P. H. Ab Initio Study of the Role of Oxygen and Excess Electrons in the Degradation of CH3NH3PbI3. J. Mater. Chem. A Mater. 2017, 5 (19), 9042–9049. 10.1039/C7TA01091E.

[ref13] Aristidou N.; Eames C.; Sanchez-molina I.; Bu X.; Kosco J.; Islam M. S.; Haque S. A. Fast Oxygen Diffusion and Iodide Defects Mediate Oxygen-Induced Degradation of Perovskite Solar Cells. Nat. Commun. 2017, 8 (1), 15218 10.1038/ncomms15218. 28492235 PMC5437277

[ref14] He J.; Fang W. H.; Long R.; Prezhdo O. V. Superoxide/Peroxide Chemistry Extends Charge Carriers’ Lifetime but Undermines Chemical Stability of CH3NH3PbI3 Exposed to Oxygen: Time-Domain Ab Initio Analysis. J. Am. Chem. Soc. 2019, 141 (14), 5798–5807. 10.1021/jacs.8b13392. 30882215

[ref15] Aristidou N.; Sanchez-Molina I.; Chotchuangchutchaval T.; Brown M.; Martinez L.; Rath T.; Haque S. A. The Role of Oxygen in the Degradation of Methylammonium Lead Trihalide Perovskite Photoactive Layers. Angew. Chem. 2015, 127 (28), 8326–8330. 10.1002/ange.201503153. 26014846

[ref16] An Y.; Perini C. A. R.; Hidalgo J.; Castro-Méndez A. F.; Vagott J. N.; Li R.; Saidi W. A.; Wang S.; Li X.; Correa-Baena J. P. Identifying High-Performance and Durable Methylammonium-Free Lead Halide Perovskites: Via High-Throughput Synthesis and Characterization. Energy Environ. Sci. 2021, 14 (12), 6638–6654. 10.1039/D1EE02691G.

[ref17] Ouyang Y.; Shi L.; Li Q.; Wang J. Role of Water and Defects in Photo-Oxidative Degradation of Methylammonium Lead Iodide Perovskite. Small Methods 2019, 3 (7), 2–7. 10.1002/smtd.201900154.

[ref18] Rajendra Kumar G.; Dennyson Savariraj A.; Karthick S. N.; Selvam S.; Balamuralitharan B.; Kim H. J.; Viswanathan K. K.; Vijaykumar M.; Prabakar K. Phase Transition Kinetics and Surface Binding States of Methylammonium Lead Iodide Perovskite. Phys. Chem. Chem. Phys. 2016, 18 (10), 7284–7292. 10.1039/C5CP06232B. 26894928

[ref19] Godding J. S. W.; Ramadan J. A.; Lin Y.-H.; Schutt K.; Snaith H. J.; Wenger B. Oxidative Passivation of Metal Halide Perovskites. Joule 2019, 3 (11), 2716–2731. 10.1016/j.joule.2019.08.006.

[ref20] Zabilska A.; Clark A. H.; Ferri D.; Nachtegaal M.; Kröcher O.; Safonova O. V. Beware of beam damage under reaction conditions: X-ray induced photochemical reduction of supported VO_*x*_ catalysts during *in situ* XAS experiments. Phys. Chem. Chem. Phys. 2022, 24 (36), 21916–21926. 10.1039/D2CP02721F. 36069029 PMC9641748

[ref21] Hoye R. L. Z.; Schulz P.; Schelhas L. T.; Holder A. M.; Stone K. H.; Perkins J. D.; Vigil-Fowler D.; Siol S.; Scanlon D. O.; Zakutayev A.; Walsh A.; Smith I. C.; Melot B. C.; Kurchin R. C.; Wang Y.; Shi J.; Marques F. C.; Berry J. J.; Tumas W.; Lany S.; Stevanović V.; Toney M. F.; Buonassisi T. Perovskite-Inspired Photovoltaic Materials: Toward Best Practices in Materials Characterization and Calculations. Chem. Mater. 2017, 29 (5), 1964–1988. 10.1021/acs.chemmater.6b03852.

[ref22] Gratia P.; Zimmermann I.; Schouwink P.; Yum J. H.; Audinot J. N.; Sivula K.; Wirtz T.; Nazeeruddin M. K. The Many Faces of Mixed Ion Perovskites: Unraveling and Understanding the Crystallization Process. ACS Energy Lett. 2017, 2 (12), 2686–2693. 10.1021/acsenergylett.7b00981.

[ref23] Muhammad Z.; Liu P.; Ahmad R.; Jalali-Asadabadi S.; Franchini C.; Ahmad I. Revealing the Quasiparticle Electronic and Excitonic Nature in Cubic, Tetragonal, and Hexagonal Phases of FAPbI3. AIP Adv. 2022, 12 (2), 025330 10.1063/5.0076738.

[ref24] Park B.-W.; Kwon H. W.; Lee Y.; Lee D. Y.; Kim M. G.; Kim G.; Kim K.-j.; Kim Y. K.; Im J.; Shin T. J.; Seok S. Stabilization of formamidinium lead triiodide α-phase with isopropylammonium chloride for perovskite solar cells. Nat. Energy 2021, 6 (4), 419–428. 10.1038/s41560-021-00802-z.

[ref25] Siegler T. D.; Dunlap-Shohl W. A.; Meng Y.; Yang Y.; Kau W. F.; Sunkari P. P.; Tsai C. E.; Armstrong Z. J.; Chen Y. C.; Beck D. A. C.; Meilă M.; Hillhouse H. W. Water-Accelerated Photooxidation of CH3NH3PbI3 Perovskite. J. Am. Chem. Soc. 2022, 144 (12), 5552–5561. 10.1021/jacs.2c00391. 35296136

[ref26] Moot T.; Dikova D. R.; Hazarika A.; Schloemer T. H.; Habisreutinger S. N.; Leick N.; Dunfield S. P.; Rosales B. A.; Harvey S. P.; Pfeilsticker J. R.; Teeter G.; Wheeler L. M.; Larson B. W.; Luther J. M. Beyond Strain: Controlling the Surface Chemistry of CsPbI3Nanocrystal Films for Improved Stability against Ambient Reactive Oxygen Species. Chem. Mater. 2020, 32 (18), 7850–7860. 10.1021/acs.chemmater.0c02543.

[ref27] Moulder J. F.; Stickle W. F.; Sobol P. E.; Bomber K. D. Handbook of X-ray Photoelectron Spectroscopy, 1995th ed.; Chastain J., King R. C., Eds.; Physical Electronics, Inc.: Eden Praire, MN, 1992.

[ref28] Lahiri N.; Song D.; Zhang X.; Huang X.; Stoerzinger K. A.; Carvalho O. Q.; Adiga P. P.; Blum M.; Rosso K. M. Interplay between Facets and Defects during the Dissociative and Molecular Adsorption of Water on Metal Oxide Surfaces. J. Am. Chem. Soc. 2023, 145 (5), 2930–2940. 10.1021/jacs.2c11291. 36696237

[ref29] Nakamura Y.; Shibayama N.; Fujiwara K.; Koganezawa T.; Miyasaka T. Degradation Mechanism of Halide Perovskite Crystals under Concurrent Light and Humidity Exposure. ACS Mater. Lett. 2022, 4 (12), 2409–2414. 10.1021/acsmaterialslett.2c00744.

[ref30] Cheng S.; Zhong H. What Happens When Halide Perovskites Meet with Water?. J. Phys. Chem. Lett. 2022, 13 (10), 2281–2290. 10.1021/acs.jpclett.2c00166. 35244396

[ref31] Mosconi E.; Azpiroz J. M.; De Angelis F. Ab Initio Molecular Dynamics Simulations of Methylammonium Lead Iodide Perovskite Degradation by Water. Chem. Mater. 2015, 27 (13), 4885–4892. 10.1021/acs.chemmater.5b01991.

[ref32] Caddeo C.; Saba M. I.; Meloni S.; Filippetti A.; Mattoni A. Collective Molecular Mechanisms in the CH3NH3PbI3 Dissolution by Liquid Water. ACS Nano 2017, 11 (9), 9183–9190. 10.1021/acsnano.7b04116. 28783296

[ref33] Frost J. M.; Butler K. T.; Brivio F.; Hendon C. H.; Van Schilfgaarde M.; Walsh A. Atomistic Origins of High-Performance in Hybrid Halide Perovskite Solar Cells. Nano Lett. 2014, 14 (5), 2584–2590. 10.1021/nl500390f. 24684284 PMC4022647

[ref34] Raval P.; Kennard R. M.; Vasileiadou E. S.; Dahlman C. J.; Spanopoulos I.; Chabinyc M. L.; Kanatzidis M.; Manjunatha Reddy G. N. Understanding Instability in Formamidinium Lead Halide Perovskites: Kinetics of Transformative Reactions at Grain and Subgrain Boundaries. ACS Energy Lett. 2022, 7 (4), 1534–1543. 10.1021/acsenergylett.2c00140.

[ref35] Caddeo C.; Marongiu D.; Meloni S.; Filippetti A.; Quochi F.; Saba M.; Mattoni A. Hydrophilicity and Water Contact Angle on Methylammonium Lead Iodide. Adv. Mater. Interfaces 2019, 6 (3), 1801173 10.1002/admi.201801173.

[ref36] Meggiolaro D.; Mosconi E.; De Angelis F. Mechanism of Reversible Trap Passivation by Molecular Oxygen in Lead-Halide Perovskites. ACS Energy Lett. 2017, 2 (12), 2794–2798. 10.1021/acsenergylett.7b00955.

[ref37] Walsh A.; Scanlon D. O.; Chen S.; Gong X. G.; Wei S. H. Self-Regulation Mechanism for Charged Point Defects in Hybrid Halide Perovskites. Angew. Chem., Int. Ed. 2015, 54 (6), 1791–1794. 10.1002/anie.201409740. PMC4344816 25504875

[ref38] Wei J.; Wang Q.; Huo J.; Gao F.; Gan Z.; Zhao Q.; Li H. Mechanisms and Suppression of Photoinduced Degradation in Perovskite Solar Cells. Adv. Energy Mater. 2021, 11 (3), 1–31. 10.1002/aenm.202002326.

[ref39] Christians J. A.; Schulz P.; Tinkham J. S.; Schloemer T. H.; Harvey S. P.; Tremolet De Villers B. J.; Sellinger A.; Berry J. J.; Luther J. M. Tailored interfaces of unencapsulated perovskite solar cells for >1,000 hour operational stability. Nat. Energy 2018, 3 (1), 68–74. 10.1038/s41560-017-0067-y.

[ref40] Yang S.; Chen S.; Mosconi E.; Fang Y.; Xiao X.; Wang C.; Zhou Y.; Yu Z.; Zhao J.; Gao Y.; De Angelis F.; Huang J. Stabilizing Halide Perovskite Surfaces for Solar Cell Operation with Wide-Bandgap Lead Oxysalts. Science (1979) 2019, 365, 473–478. 10.1126/science.aax3294. 31371610

[ref41] Oner S. M.; Sezen E.; Yordanli M. S.; Karakoc E.; Deger C.; Yavuz I. Surface Defect Formation and Passivation in Formamidinium Lead Triiodide (FAPbI3) Perovskite Solar Cell Absorbers. J. Phys. Chem. Lett. 2022, 13 (1), 324–330. 10.1021/acs.jpclett.1c03645. 34978837

[ref42] Jiang Q.; Zhao Y.; Zhang X.; Yang X.; Chen Y.; Chu Z.; Ye Q.; Li X.; Yin Z.; You J. Surface Passivation of Perovskite Film for Efficient Solar Cells. Nat. Photonics 2019, 13 (7), 460–466. 10.1038/s41566-019-0398-2.

[ref43] Perini C. A. R.; Rojas-Gatjens E.; Ravello M.; Castro-Mendez A. F.; Hidalgo J.; An Y.; Kim S.; Lai B.; Li R.; Silva-Acuña C.; Correa-Baena J. P. Interface Reconstruction from Ruddlesden-Popper Structures Impacts Stability in Lead Halide Perovskite Solar Cells. Adv. Mater. 2022, 34 (51), 2204726 10.1002/adma.202204726. 36245328

[ref44] Li N.; Luo Y.; Chen Z.; Niu X.; Zhang X.; Lu J.; Kumar R.; Jiang J.; Liu H.; Guo X.; Lai B.; Brocks G.; Chen Q.; Tao S.; Fenning D. P.; Zhou H. Microscopic Degradation in Formamidinium-Cesium Lead Iodide Perovskite Solar Cells under Operational Stressors. Joule 2020, 4 (8), 1743–1758. 10.1016/j.joule.2020.06.005.

