# Supplementary material for: Synergistic Role of Water and Oxygen Leads to Degradation in Formamidinium-Based Halide Perovskites
Source: J Am Chem Soc. 2023 Nov 2;145(45):24549–57. doi: 10.1021/jacs.3c05657 (PMC10655111; doi:10.1021/jacs.3c05657)
Supplement: Supplementary file 1 — ja3c05657_si_001.pdf [file ja3c05657_si_001.pdf]

Supplementary Information

Synergistic Role of Water and Oxygen Leads to Degradation in Formamidinium-Based Halide Perovskites

Juanita Hidalgo [1], Waldemar Kaiser [2], Yu An [1], Ruipeng Li [3], Zion Oh [1], Andrés-Felipe Castro-Méndez [1], Diana K. LaFollette [1], Sanggyun Kim [1], Barry Lai [4], Joachim Breternitz [5], Susan Schorr [5,6], Carlo A.R Perini [1], Edoardo Mosconi [2], Filippo De Angelis [2,7,8,9], Juan-Pablo Correa-Baena [1*]

[1] School of Materials Science and Engineering, Georgia Institute of Technology, Atlanta, Georgia 30332, United States.

[2] Computational Laboratory for Hybrid/Organic Photovoltaics (CLHYO), Istituto CNR di Scienze e Tecnologie Chimiche "Giulio Natta" (CNR-SCITEC), 06123 Perugia, Italy.

[3] National Synchrotron Light Source II, Brookhaven National Lab, Upton, New York, 11973, United States.

[4] Advanced Photon Source, Argonne National Laboratory, Lemont, Illinois, 60439, United States.

[5] Department of Structure and Dynamics of Energy Materials, Helmholtz-Zentrum Berlin für Materialien und Energie, Hahn-Meitner-Platz 1, 14109, Berlin, Germany.

[6] Freie Universitaet Berlin, Institute of Geological Sciences, Malteser Str. 74-200, 12249 Berlin, Germany.

[7] Department of Chemistry, Biology and Biotechnology, University of Perugia and UdR INSTM, 06123 Perugia, Italy.

[8]Department of Natural Sciences & Mathematics, College of Sciences & Human Studies, Prince Mohammad Bin Fahd University, Dhahran 34754, Saudi Arabia.

[9]SKKU Institute of Energy Science and Technology (SIEST), Sungkyunkwan University, Suwon 440-746, Korea.

*Corresponding Authors: JPCB jpcorrea@gatech.edu

1. Methodology

1.1 Experimental

Perovskite thin film fabrication: $Cs_{0.17}FA_{0.83}PbI_3$ (CsFA) was prepared from precursor powders PbI_2 (Tokyo Chemical Industry, > 99.99 %), FAI (Great Solar), and CsI (Sigma-Aldrich) in a mixture of anhydrous dimethyl formamide (DMF, Sigma Aldrich) and dimethyl sulfoxide (DMSO, Acros Organics) in a volume ratio of 2:1 DMF:DMSO to form a 1.2M solution. The solution stoichiometry had a 5% molar excess of PbI_2. The CsFA solution was mixed for two hours, and then was spin coated first at 1000 rpm for 10 s, followed by 6000 rpm for 20 s and adding chlorobenzene (Sigma Aldrich) 5 s before the end of the spin coating process. The deposited CsFA films were annealed at 150 °C for 15 min.

Ex-situ humidity: The CsFA films were deposited in a nitrogen glove box. After the annealing step, the films were taken to a box with a relative humidity of ~80% in air. For the *ex-situ* experiments, the films were kept for 20 hours in the humid air box in dark.

Powder synthesis: CsI (Sigma Aldrich), FAI (TCI), and PbI_2 (TCI) powders were mixed in 3 mL of gamma butyrolactone (GBL, TCI) and dimethyl formamide (DMF, TCI) with a volume ratio of 2:1 to form a 0.5M solution of $Cs_{0.17}FA_{0.83}PbI_3$, with 5% molar excess of PbI_2. The solution was mixed for 1 hour at 50 °C. After, the solution was evaporated in a petri-dish at 170 °C until dried and annealed for 1 hour at the same temperature.

FAI and PbI_2 thin films: FAI powder (Great Solar) or PbI_2 powder (TCI) were mixed in dimethyl formamidine (DMF) and dimethyl sulfoxide (DMSO) with a volume ratio of 2:1 volume ratio to form a 1.2M solution of FAI or PbI_2. The solution was deposited via spin coating on glass substrates following the same deposition method of CsFA perovskite (1000rpm for 10s, and 6000rpm for 20s). Chlorobenzene was added as antisolvent 5 s before the end of the second spin coating step. The films were annealed at 100 °C for 15 min.

Solar cell fabrication with PEAI: Patterned fluorine-doped tin oxide (FTO) on glass substrates were cleaned by sonicating the substrates 15 min in (1) a solution of 2% Mucasol, (2) deionized water, (3) acetone, and (4) isopropyl alcohol (IPA). As electron transport layer, a compact layer of TiO_2 (c-TiO_2) was deposited by spray pyrolysis at 450 °C from a solution containing 480 μL acetylacetone (Sigma-Aldrich, ≥ 99%), 720 μL titanium diisopropoxide bis(acetylacetonate) 75 wt. % in isopropanol (Sigma-Aldrich), and 10.8 mL of ethanol (Sigma-Aldrich, ≥ 99.5%). The solution was sprayed using O_2 as carrier gas, in cycles with 30 s intervals until finishing the solution. The c-TiO_2 was annealed for 30 min. Subsequently, a mesoporous TiO_2 layer (mp-TiO_2) was deposited by spin coating a TiO_2 paste (Sigma Aldrich) in ethanol (150 mg/mL) for 10 s at 4000 rpm and annealed for 10 min at 100 °C. The mp-TiO_2 layer followed a sintering process found in other articles [1] reaching 450 °C for 30 min. After, inside the nitrogen glovebox, the CsFA perovskite layer was deposited by spin coating. For the PEAI-treated substrates, a PEAI solution with a concentration of 5 mg/mL in IPA (99.9% Sigma Aldrich), was spin coated for 20 s at 5000 rpm. For the ex-situ exposure to humidity, after the PEAI layer, the films are exposed to humid air for 20 hours. After exposure to humidity, the samples are taken to the nitrogen glovebox to immediately deposit the missing layers in nitrogen. Subsequently, we prepare the hole transport layer solution by mixing Spiro-OMeTAD (1-Material) 28.4 M in chlorobenzene, adding 0.4 mol

per mol of Spiro of a solution of LiTFSI (Sigma Aldrich, 1.8 M in acetonitrile), 3.3 mol per mol of Spiro of 4-tertbutilpiridine (Sigma Aldrich), and 0.03 mol per mol of Spiro of a solution of Co (II) salt (FK209, Sigma Aldrich, 0.25M in acetonitrile). The hole transport layer solution is spin coated dynamically at 3000 rpm for 30 s. Finally, 50nm of Au (Kurt Lesker) is thermally evaporated to finish the device.

1.2 Characterization

In-situ GIWAXS: The GIWAXS measurements were performed at the beamline for Complex Materials Scattering (11-BM) at the National Synchrotron Light Source II at Brookhaven National Laboratory. The perovskite films for *in-situ* GIWAXS were deposited on glass/ITO or glass/FTO/TiO_2. The X-ray beam had an energy of 13.5 keV, with a footprint of 0.2 mm × 0.05 mm. The samples were irradiated for 10 s with an incident angle of 0.1° and 0.5°. Beam divergence was 1 mrad and energy resolution 0.7%. The data were analyzed using the SciAnalysis package provided by the beamline.

For the *in-situ* measurements, we used a controlled relative humidity chamber (**Figure S1**). For a relative humidity of 100% all the gas into the chamber came from a water bubbler. The humidity carrier gas into the water bubbler was either dry air or nitrogen. A controller was used to monitor the relative humidity inside the measurement chamber as the experiments took place. For the *Dry Air* experiment, the same line of dry air was sent directly to the measurement chamber. The room light was on during the whole experiment.

In-situ setup details:

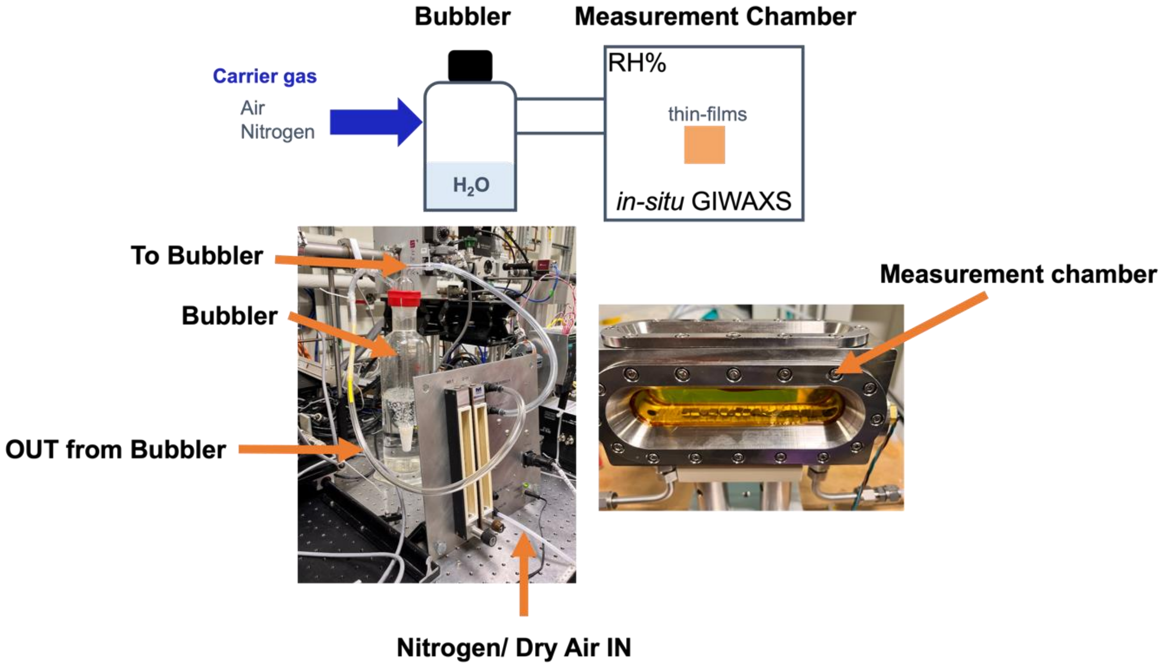

Figure S1. Experimental setup for the *in-situ* relative humidity GIWAXS measurements at BNL NSLS II, CMS 11-BM.

X-ray Photoelectron Spectroscopy (XPS): XPS was measured with a Thermo Scientific K-Alpha system using a monochromatic Al K-alpha X-ray source (1486.6 eV) with a 60° incident angle and a 0° photoemission angle, both measured from the samples normal vector. The pressure of the chamber was less than 1e^{-7} Torr. Survey and high-resolution scans were collected. High-resolution scans were taken with a step size of 0.100 eV and a dwell time of 50 ms for C1s (20 scans), N1s (16 scans), I3d (3 scans), O1s (20 scans), Pb4f (3 scans), and Cs3d (3 scans). Peak fitting and elemental composition were calculated with the Thermo Scientific Avantage Data System.

XRD: Powder X-ray diffraction measurements were done in ambient in a Bruker D8 instrument with a Bragg-Brentano geometry. The Le-Bail analysis was done using the FullProf software, and the structure identification using CrystalMaker software.

UV-VIS: UV-VIS was done in a PerkinElmer Lambda 1050 spectrophotometer and steady state PL in the Horiba FL3-21 fluorometer.

TRPL: TRPL decays were obtained using a 405 nm pulsed laser (LDH-D-C-405, PicoQuant) at a repetition frequency of 125 kHz and a power density of 4.1 mW/cm^2. A 550 nm long pass filter (ThorLabs) was used to removed excess laser scatter. Photon arrival times were recorded using a single-photon avalanche photodiode (Micro Photon Devices) connected to a MultiHarp 150 event timer (PicoQuant). Incident laser powers were measured with a silicon power meter (ThorLabs PM100-D) and spot sizes were measured using the razor blade method (90:10).

SEM: Film morphology was characterized using a SEM Hitachi SU8010 1nm resolution and secondary electron detector at 5keV and 10uA.

XRF-XBIC: Synchrotron XRF and XBIC measurements were done to CsFA films on glass for only XRF measurements, and to complete perovskite solar cell devices for XRF-XBIC measurements, at beamline 2-ID-D at the Advanced Photon Source (Argonne National Laboratory), with a synchrotron X-ray of energy 16.5keV and a step size of 0.15um and 50ms dwell time. For the XRF-XBIC, the measurement was performed with the Au contact facing the incident X-ray beam, with fluorescence spectrum collected point-by-point during mapping. Data was obtained in the same area for fluorescence and induced current in arbitrary units of intensity, giving a direct correlation between XRF and XBIC. The MAPS software was used for data analysis and spectrum fitting to deconvolute overlapping peaks and background from fluorescence data. In addition, after a standard calibration, it was possible to quantify in the software the mass concentration in the sample to calculate accurately the ratio between Cs:Pb. The NIST thin-film standards SRM 1832 and 1833 were used for calibration of the elemental concentrations.

FTIR powder: A FTIR Spectrometer from Agilent Technologies Cary 630 with Ge-ATR, with a resolution of 4cm^{-1} was used.

FTIR films: A Nicolet 5 FTIR Spectrometer was used to measure FTIR on the thin films.

Solar cells and stability: The current density- voltage (*J-V*) characteristics of the solar cells were

measured using a LITOS LITE setup (Fluxim, Switzerland) equipped with a Wavelabs Sinus-70 AAA solar simulator with an illumination of AM 1.5 G at room temperature and ambient air. The *J-V* curves were measured in a range from 1.4 to -0.5 V with a scan speed of 50 mVs^{-1}. The active area of the device was 0.128 cm^2 and a mask of 0.0625cm^2 was used. For the long-term stability measurement, we used LITOS, a stress-test platform for degradation analysis (Fluxim, Switzerland). The solar cells were stressed by 1 sun illumination and introducing a constant flow of air. The power density was measured every 100 seconds, for 24 hours, without mask.

1.3 First Principles Calculations Methods

<u>Surface models and computational details</u>

Density functional theory (DFT) calculations of the interaction of oxygen and water molecules with perovskite surfaces were performed within the Quantum Espresso software package.[2] Geometry optimization of the slab models were performed using the PBE exchange-correlation functional [3] with plane-wave basis set cutoffs for the smooth part of the wave functions and augmented electronic density expansions of 40 and 320 Ry, respectively. Dispersion interactions are captured within the DFT-D3 scheme.[4] Electron-ion interactions are described by ultrasoft, scalar-relativistic pseudo-potentials with electrons from O, N, and C 2s, 2p; H 1s; I 5s, 5p; Cs 5s, 5p, 6s; and Pb 6s, 6p, and 5d shells explicitly included in the calculations. The cell is generated starting from the β-phase of $FAPbI_3$ [5], with subsequent replacing of 6 of 32 FA^+ by Cs^+ cations within a 2×2×2 supercell, resulting in a $Cs_{0.19}FA_{0.81}PbI_3$ composition. Subsequent geometry and cell optimization, keeping angles fixed, are performed before generation of the slab models. The PbI_2- and $Cs_xFA_{1-x}I$-terminated perovskite slab models were made of 5 inorganic layers and of 3 inorganic layers, respectively, as shown in **Figure S17**. A vacuum region of at least 15 Å was added on top of each surface. For the calculation of water adsorption on PEA cations, we created a PEA_2PbI_4 slab model made of 2 inorganic layers with PEAI-termination and >15 Å vacuum within a 2×2 supercell along a and b direction, using experimental cell parameters.[6] In all calculations, the Brillouin zone is sampled at Γ-point.

<u>Modeling of Reactions</u>

Rx.1: The formation energies of oxidized iodide species, IO_n^-, are calculated as follows:

$$\Delta E = E(\text{slab} + IO_n^-) - E(\text{slab}) - \frac{n}{2}E(O_2)$$

where $E(\text{slab} + IO_n^-)$ is the energy of the surface model containing the IO_n^-, E(slab) is the energy of the pristine slab, and $E(O_2)$ is the energy of an oxygen molecule.

Rx.2: The formation energy of lead(II) iodate, $Pb(IO_3)_2$, is calculated as follows:

$$\Delta E = E(\text{slab} + Pb(IO_3)_2) - E(\text{slab}) - 3E(O_2)$$

where $E(\text{slab} + Pb(IO_3)_2)$ is the energy of the surface model containing the $Pb(IO_3)_2$.

Rx.3: The formation energies of a PbI_2 vacancy upon removal of a $Pb(IO_3)_2$ unit is calculated as follows:

$$\Delta E = E(\text{def.}) - E(\text{slab} + Pb(IO_3)_2) + \mu(Pb(IO_3)_2)$$

where $E(\text{def.})$ is the defective surface the PbI_2 vacancy, and $\mu(Pb(IO_3)_2)$ is the chemical potential of lead (II) iodate. To obtain the chemical potential, we calculated the energy of a bulk lead (II)

iodate [7] available at CCDC 1635205. Geometry optimization of the lead (II) iodate phase were done using high 8×8×1 k-point sampling and D3 corrections at the PBE level of theory.

DFT model of hydration

DFT calculations of the oxygen molecular orbitals are performed using the Gaussian09 program package.[8] Geometry optimization in water was done using the C-PCM model [9] using explicit solvation by four water molecules. All quantities are calculated employing the hybrid B3LYP functional [10] along with 6-311g** basis set and D3 corrections.

2. Results and Discussion

2.1 Structural phase transformations

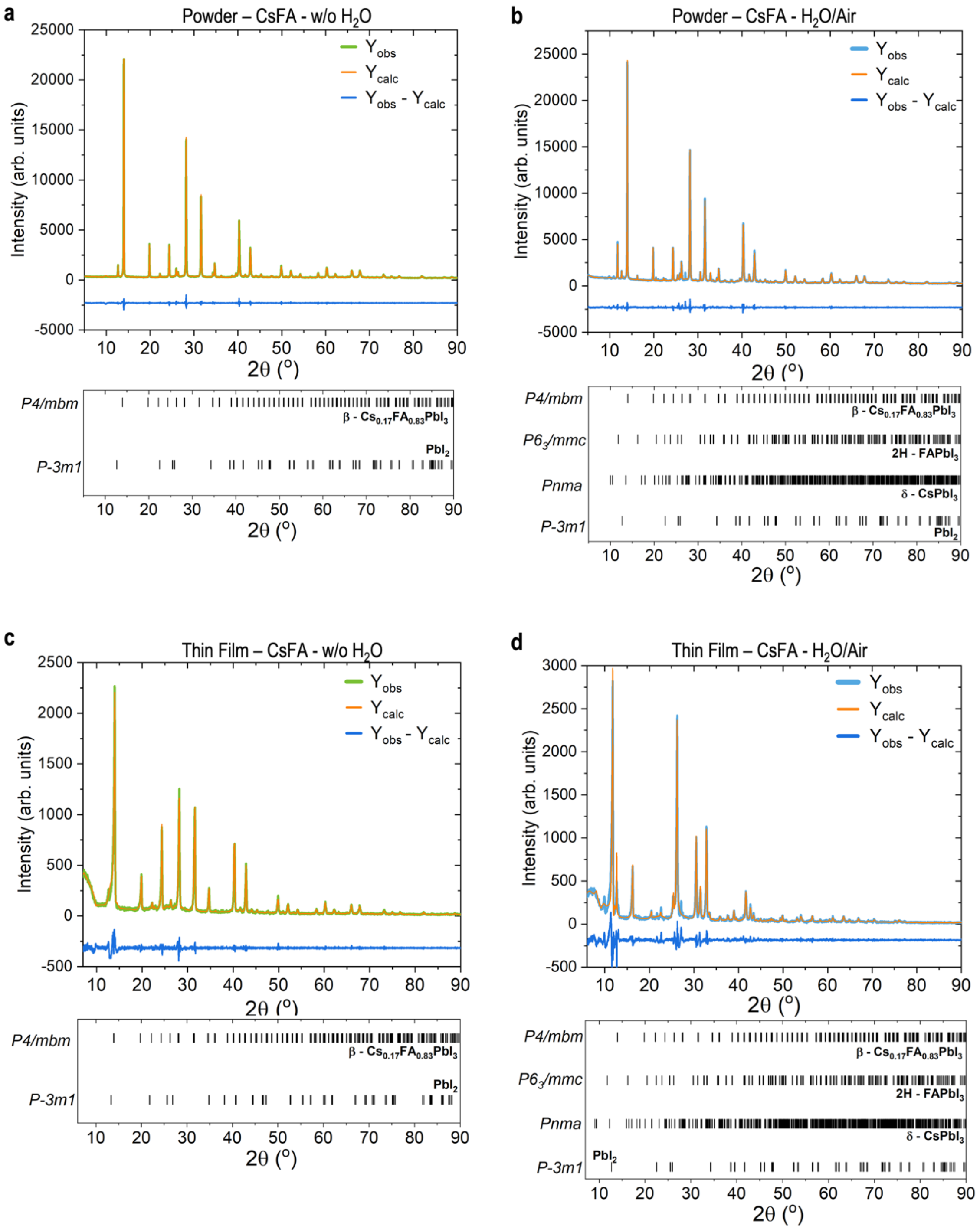

Figure S2. Le Bail analysis of powder diffraction and thin-film diffraction data. **a)** $Cs0._{17}FA_{0.83}PbI_3$ powders without (w/o) H_2O exposure, and **b)** powders exposed *ex-situ* to H_2O/Air with high relative humidity of 80% for 24 hours **c)** $Cs_{0.17}FA_{0.83}PbI_3$ thin films without (w/o) H_2O exposure, and **d)** exposed *ex-situ* to H_2O/Air with high relative humidity of 80% for 20 hours.

Table S1. Lattice parameters from LeBail analysis in Fig. S2

Phase	Space group	Lattice parameters						
		a / Å	b / Å	c / Å	alpha /°	beta /°	gamma /°	Chi^2
**Thin Film w/o H_2O**								
1. Perovskite	*P4/mbm*	8.95	8.95	6.36	90	90	90	1.29
2. PbI_2	$P\bar{3}m1$	4.70	4.70	6.63	90	90	120	
**Thin Film H_2O/Air**								
1. Perovskite	*P4/mbm*	8.95	8.95	6.34	90	90	90	3.03
2. 2H	*P63/mmc*	8.68	8.68	7.94	90	90	120	
3. δCs	*Pnma*	10.76	3.93	19.54	90	90	90	
4. PbI_2	$P\bar{3}m1$	4.56	4.56	6.99	90	90	120	
**Powder w/o H_2O**								
1. Perovskite	*P4/mbm*	8.95	8.95	6.33	90	90	90	1.64
2. PbI_2	$P\bar{3}m1$	4.56	4.56	6.99	90	90	120	
**Powder H_2O/Air**								
1. Perovskite	*P4/mbm*	8.95	8.95	6.34	90	90	90	2.62
2. 2H	*P63/mmc*	8.68	8.68	7.93	90	90	120	
3. δCs	*Pnma*	10.37	4.17	16.92	90	90	90	
4. PbI_2	$P\bar{3}m1$	4.56	4.56	6.99	90	90	120	

To quantitatively compare the degradation rate of CsFA perovskite, we integrated the area of the main scattering peak for each phase. For the β-perovskite, we integrated the 110 peak at $q_r \sim 1 \pm 0.05$ Å^{-1}. For the 2H phase, we integrated the $1\bar{1}0$ peak at $q_r \sim 0.84 \pm 0.02$ Å^{-1}. For the δCs phase, we integrated the 002 peak at $q_r \sim 0.7 \pm 0.05$ Å^{-1} given that the main 212 peak of δCs at $q_r \sim 1.8$ Å^{-1} overlaps with other phases. The integrated area was plotted in **Fig. 1** of the main text. To further compare the phase transformation rate, we normalized the integrated area (α) for each phase as shown in **Fig. S3a**. We observe that the main β-perovskite phase is lost when exposed to H_2O/Air, compared to a more stable phase in H_2O/N_2. We fitted the phase transformation data into a sigmoidal Boltzmann function, observing a high R^2 value, hence a better fitting for the perovskite transformation in H_2O/Air. In addition, the lowest *dx* value from the Boltzmann fitting in **Fig. S3a** for the transformation in H_2O/Air confirms the faster decrease. Additionally, we linearize the data with a logarithmic function $[-\ln(1-\alpha)]^{1/2}$ used in other crystal transformation studies [11,12] to calculate a time constant b that corresponds to the slope of the fitted linear function. In the case of the β110 peak in **Fig. S3b** we observe a b of -1.5 for the phase transformation in H_2O/Air compared to -0.5 in H_2O/Air. However, the fitting in H_2O/N_2 has a higher error. In sum, we quantify the faster loss of β-perovskite phase in H_2O/Air.

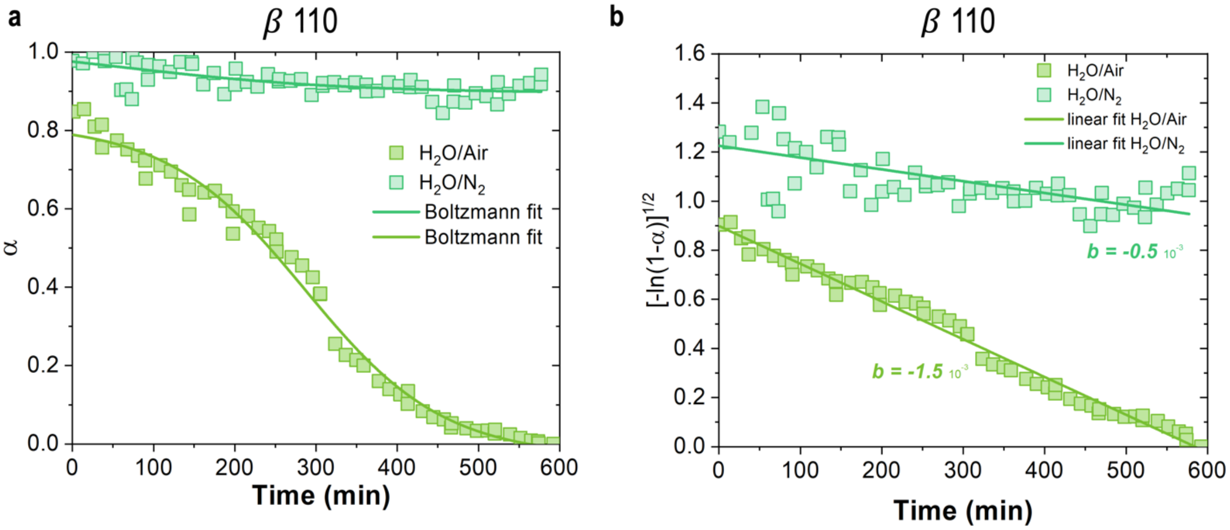

Model	Boltzmann
Equation	y = A2 + (A1-A2)/(1 + exp((x-x0)/dx))
Plot	N2
A1	1.02892 ± 0.2294
A2	0.89572 ± 0.019
x0	57.36059 ± 458.07029
dx	141.09893 ± 182.90061
Reduced Chi-Sqr	6.9242E-4
R-Square (COD)	0.47135
Adj. R-Square	0.44198

Model	Boltzmann
Equation	y = A2 + (A1-A2)/(1 + exp((x-x0)/dx))
Plot	Air
A1	0.81741 ± 0.01996
A2	-0.03265 ± 0.01805
x0	287.00294 ± 6.01686
dx	85.29884 ± 6.79125
Reduced Chi-Sqr	0.00111
R-Square (COD)	0.98808
Adj. R-Square	0.98738

Equation	y = a + b*x
Plot	N2
Weight	No Weighting
Intercept	1.2255 ± 0.02685
Slope	-4.80825E-4 ± 8.06543E-5
Residual Sum of Squares	0.60081
Pearson's r	-0.62653
R-Square (COD)	0.39254
Adj. R-Square	0.38149

Equation	y = a + b*x
Plot	Air
Weight	No Weighting
Intercept	0.89901 ± 0.00926
Slope	-0.00154 ± 2.69164E-5
Residual Sum of Squares	0.06471
Pearson's r	-0.99198
R-Square (COD)	0.98403
Adj. R-Square	0.98373

Figure S3. 110 peak evolutions from the integrated area of the β-CsFA perovskite, normalized into α, and fitted to **a)** Boltzmann sigmoidal function. **b)** Fitted into a linear logarithmic model.

We do the same analysis for the formation of the non-perovskite phases 2H and δCs by normalizing the integrated area (α) and plotting it as a function of time for the different studied conditions: **Fig. S4a** 2H phase in H_2O/Air, **Fig. S4b** 2H phase in H_2O/N_2, **Fig. S4c** δCs in H_2O/Air, and Fig. **S4d** δCs in H_2O/N_2. The non-perovskite phases in H_2O/Air form faster, showing a better Boltzmann fitting and a lower *dx* value from the fitting. Remarkably, **Fig. S4b** shows no formation of the 2H phase in H_2O/N_2. For this reason, there is no Boltzmann nor linear fitting for the data. To compare the non-perovskite phase formation under the same condition, we compared the normalized linearized data. **Fig. S4e** shows how 2H phase forms at a faster rate with a slope, time constant b, higher than for the δCs phase.

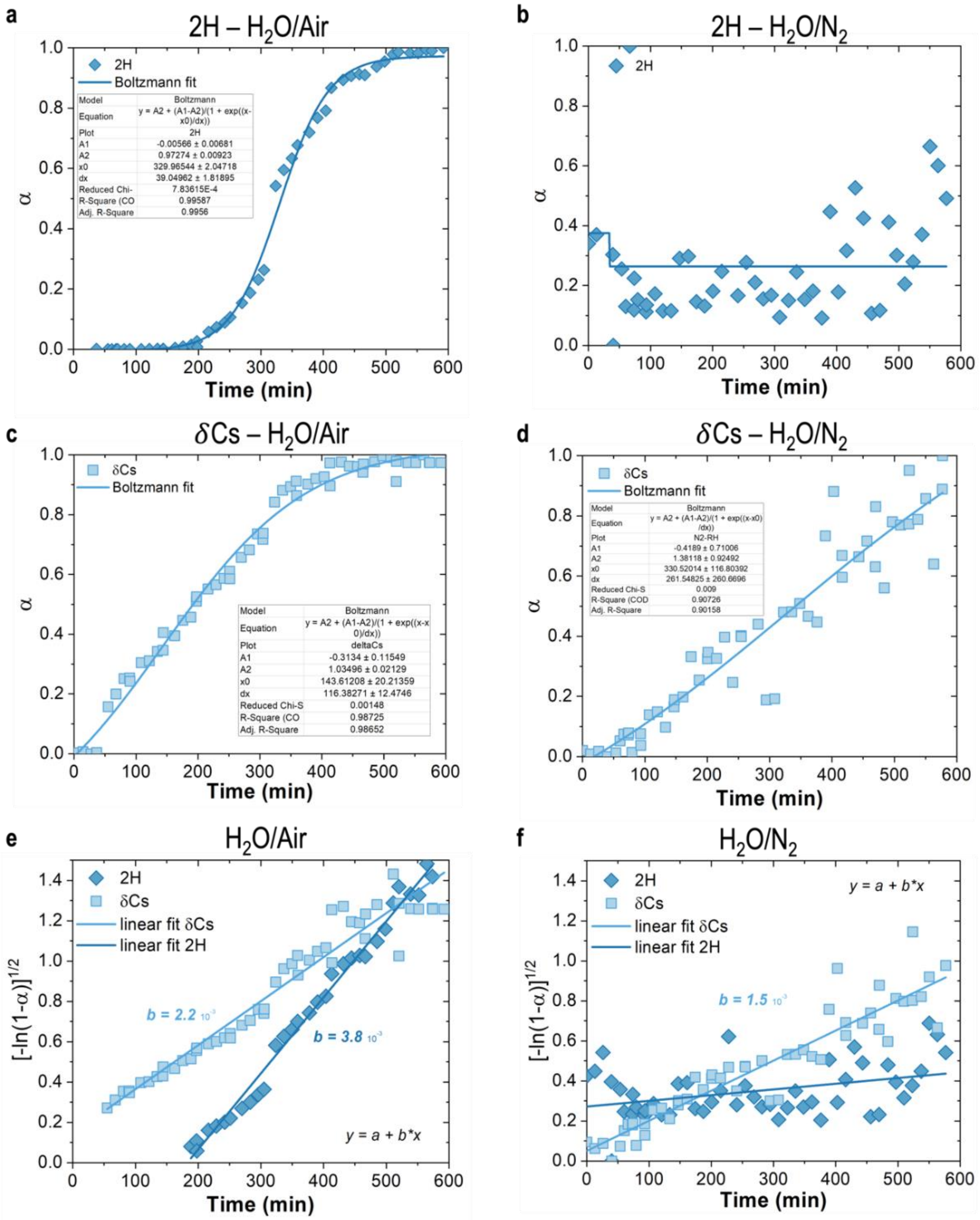

2H – H_2O/Air - linear fit

Equation	y = a + b*x
Plot	(-ln(1-a))^1/2
Weight	No Weighting
Intercept	-0.68863 ± 0.03075
Slope	0.00377 ± 7.78551E-5
Residual Sum of Squares	0.07716
Pearson's r	0.99388
R-Square (COD)	0.9878
Adj. R-Square	0.98737

δCs – H_2O/Air - linear fit

Equation	y = a + b*x
Plot	(-ln(1-a))^1/2
Weight	No Weighting
Intercept	0.14621 ± 0.0315
Slope	0.00218 ± 8.86339E-5
Residual Sum of Squares	0.45884
Pearson's r	0.96264
R-Square (COD)	0.92668
Adj. R-Square	0.92516

δCs – H_2O/N_2 - linear fit

Equation	y = a + b*x
Plot	loga
Weight	No Weighting
Intercept	0.05087 ± 0.02594
Slope	0.0015 ± 7.97631E-5
Residual Sum of Squares	0.49866
Pearson's r	0.93621
R-Square (COD)	0.87648
Adj. R-Square	0.87401

Figure S4. Peak evolution from integrated area normalized into α and fitted to a Boltzmann sigmoidal function **a)** 2H peak in H_2O/Air, **b)** 2H peak in H_2O/N_2, **c)** δCs peak in H_2O/Air, **d)** δCs peak in H_2O/N_2. Data fitted into a linear logarithmic function to compare the evolution and calculate time constant (b) in **e)** H_2O/Air and **f)** H_2O/N_2.

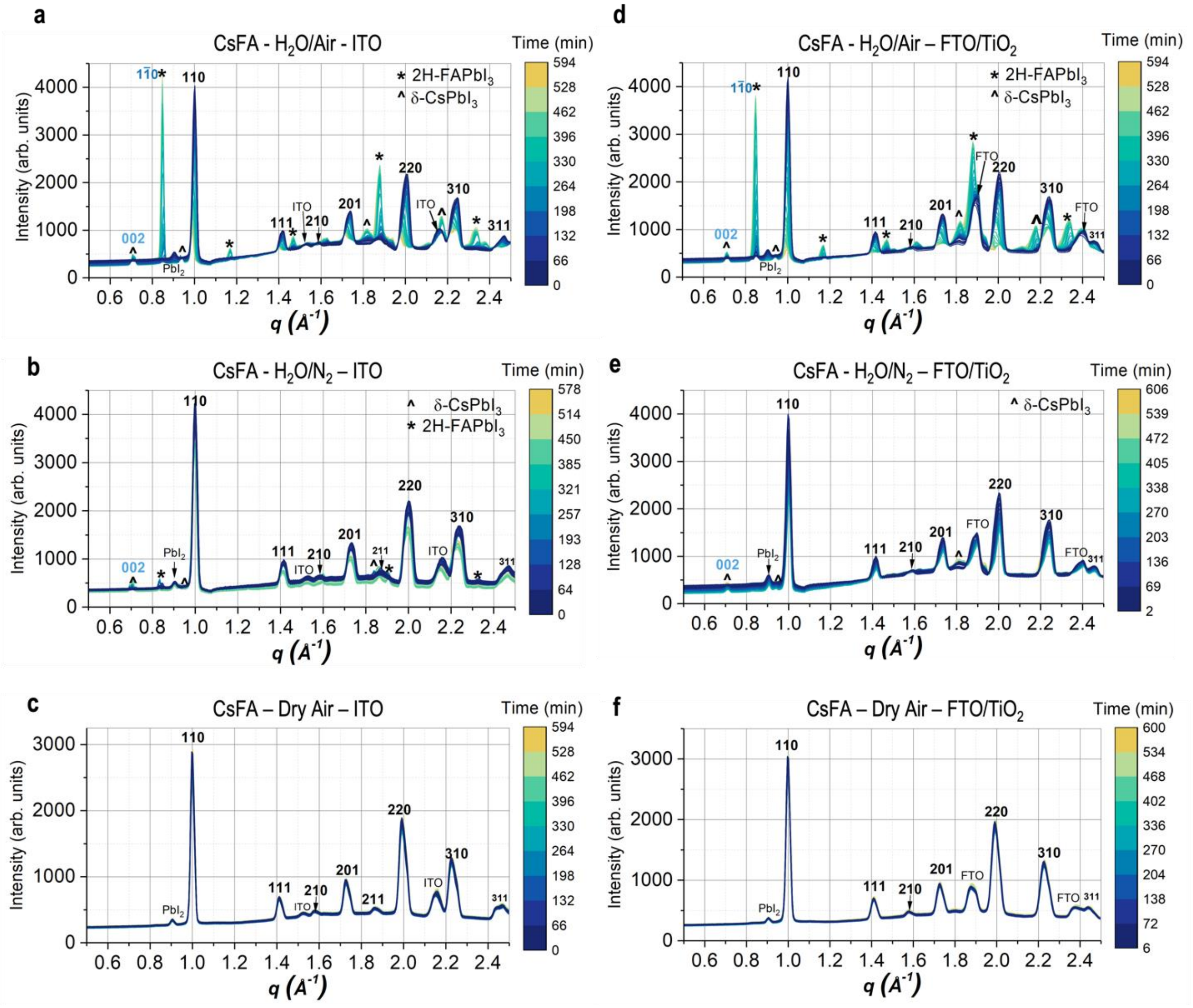

Figure S5. Integrated circular average from *in-situ* relative humidity for different substrates and exposure conditions **a)** H_2O/Air on ITO, **b)** H_2O/Nitrogen on ITO, **c)** Dry Air on ITO, **d)** H_2O/Air on FTO and compact and mesoporous TiO_2 layers, **e)** H_2O/Nitrogen on FTO and compact and mesoporous TiO_2 layers, **d)** Dry Air on FTO and TiO_2 layers. Bragg peaks are labeled for the β-perovskite phase.

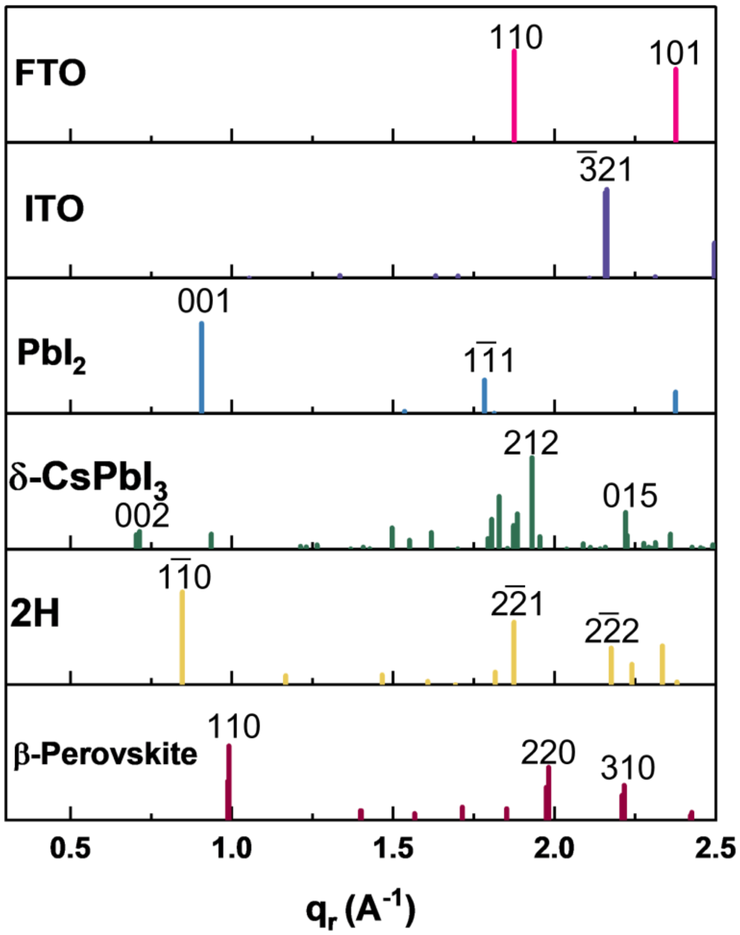

Figure S6. Peaks from simulated diffraction patters of the main phases observed in the studied lead halide perovskites: FTO, ITO, PbI_2, orthorhombic δ-$CsPbI_3$, hexagonal 2H-$FAPbI_3$, and tetragonal β-perovskite $Cs_{0.17}FA_{0.83}PbI_3$.

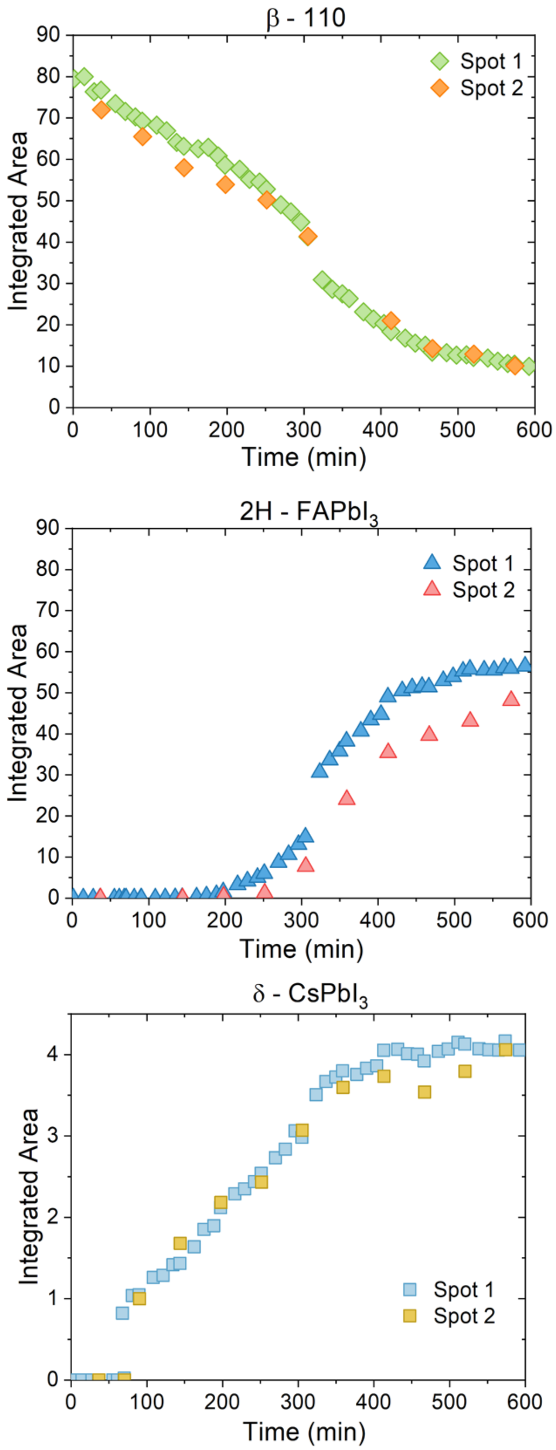

Figure S7. We measured the GIWAXS in an additional, isolated spot (spot 2), which has not seen X-rays, to make sure the peak intensity was not changing due to beam damage. Spot 2 was measured in parallel, but with less beam exposure given that the data was taken in longer time steps. Here, spot 1 is the continuous measurement as presented in **Fig. 1** of the main text.

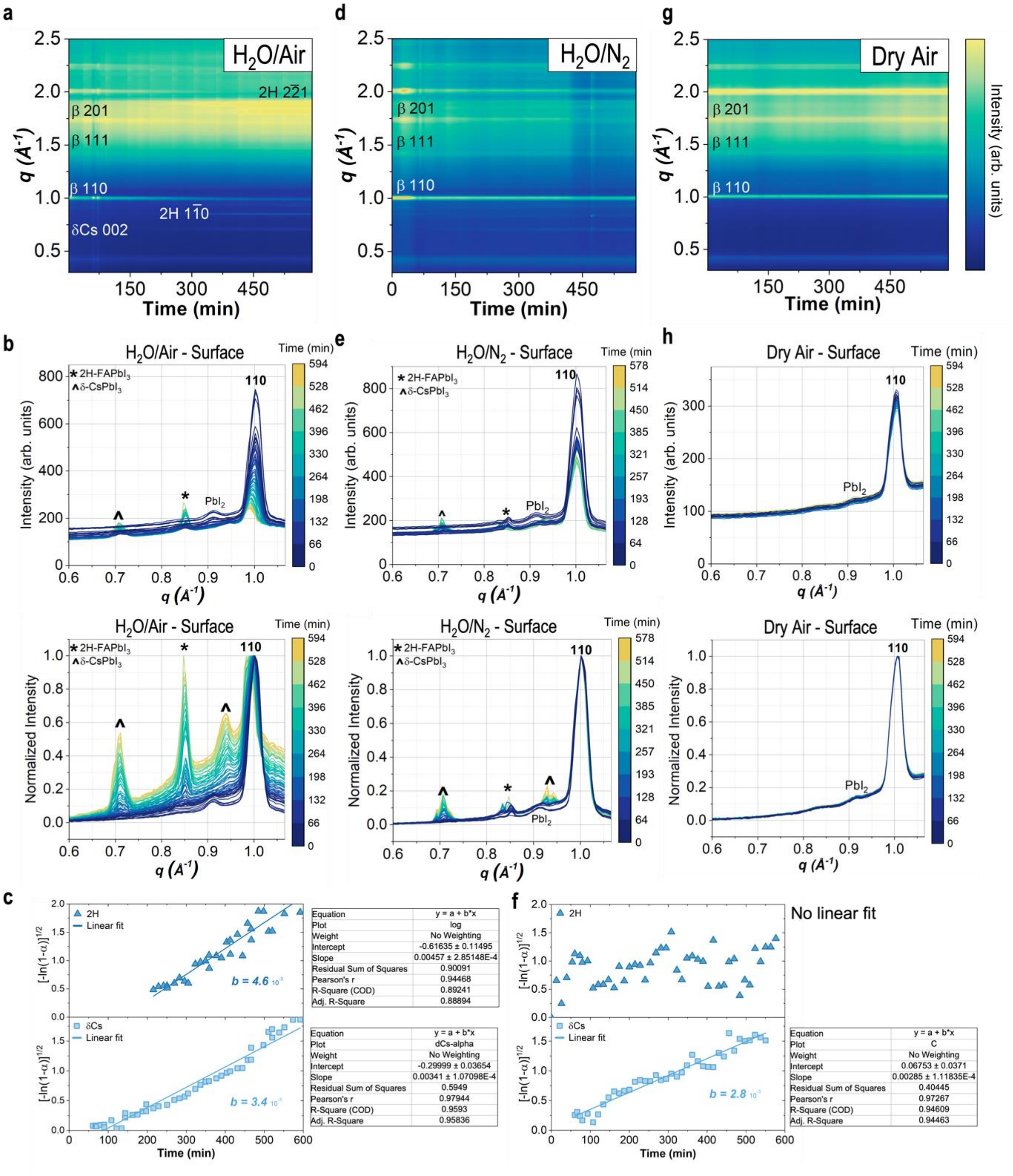

Figure S8. Structural phase analysis at the surface (GIWAXS incident angle of 0.1°, beyond the critical point for perovskites) by *in-situ* GIWAXS. **a, b, c)** CsFA films exposed to H_2O/Air, **d,e,f)** H_2O/N_2, and **g,h)** Dry air. **a, d, g)** *In-situ* scattering vector as a function of time of exposure, **b)** zoom-in plot of the circular average scattering pattern as a function of time of exposure, and the normalized-intensity plot, and **c, f)** the peak evolution of the 2H and δCs phases, following the procedure from Figs. S3 and S4, and calculating the time constant *b*.

Note on surface analysis: The surface GIWAXS is obtained from varying the X-ray incident angle, from 0.5° for bulk to 0.1° for surface. The critical angle for $FAPbI_3$ has been reported at

approximately 0.16°,[13] meaning that below we are probing only some tenths of nanometers of layer, while above, we increase to hundreds of nanometers. Given that our film has a thickness of around 500nm, the 0.5° probes the "bulk" and 0.1° probes the "surface. Herein, we observe surface phase transformations for CsFA films exposed to H_2O/Air and to H_2O/N_2. The time constant *b* shows a higher value at the surface compared to bulk (Fig. S4), indicating that the non-perovskite phases form faster on the surface than bulk. By comparing 2H with δCs in H_2O/Air, 2H phase forms faster.

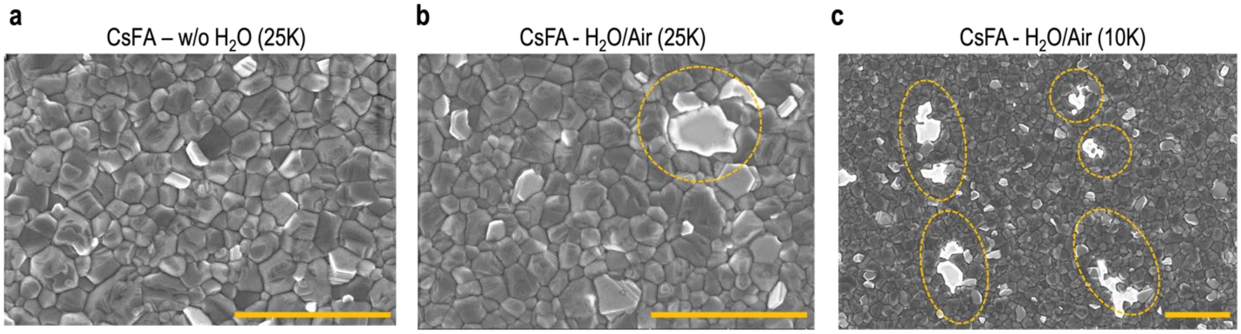

Figure S9. Morphology from SEM in **a)** $Cs_{0.17}FA_{0.83}PbI_3$ (CsFA) thin films without (w/o) H_2O exposure, and **b,c)** after exposed *ex-situ* to H_2O/Air relative humidity 80% for 20 hours. The scale bar is 2 micrometers. The SEM measurements were done in vacuum, but the samples were exposed to air from the glovebox to the SEM instrument.

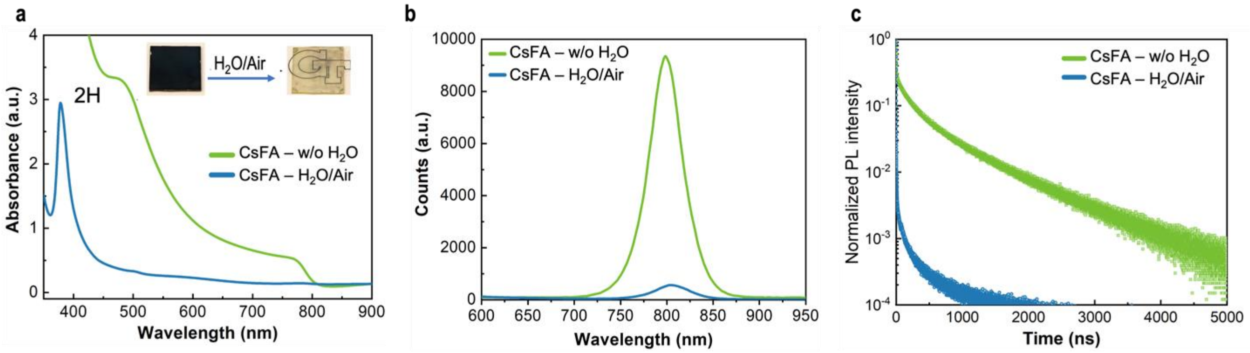

Figure S10. Optical properties of the $Cs_{0.17}FA_{0.83}PbI_3$ (CsFA) exposed *ex-situ* to H_2O/Air with relative humidity 80% for 20 hours. **a)** Absorption from UV-VIS spectroscopy, **b)** steady state photoluminescence, and **c)** transient photoluminescence with normalized PL intensity. The measurements were done in ambient atmosphere, therefore, exposed to ambient air. (green) CsFA without (w/o) H_2O exposure, (blue) after exposure to H_2O/Air

2.2 Surface chemistry

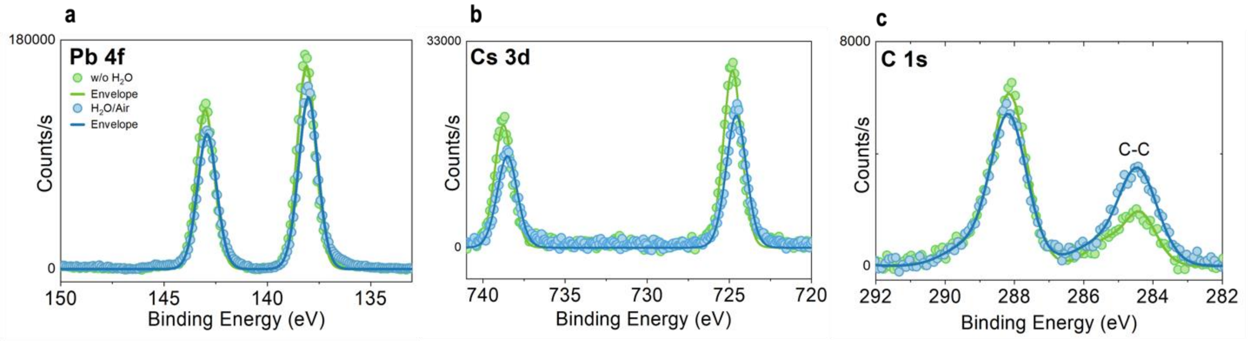

Figure S11. XPS spectra of $Cs_{0.17}FA_{0.83}PbI_3$ (CsFA) films without (w/o) exposure to H_2O. The films were deposited inside of nitrogen glovebox but exposed few minutes to air while transferring to the XPS instrument. XPS measurement was in vacuum. **a)** Pb 4f, **b)** Cs 3d, **c)** C 1s.

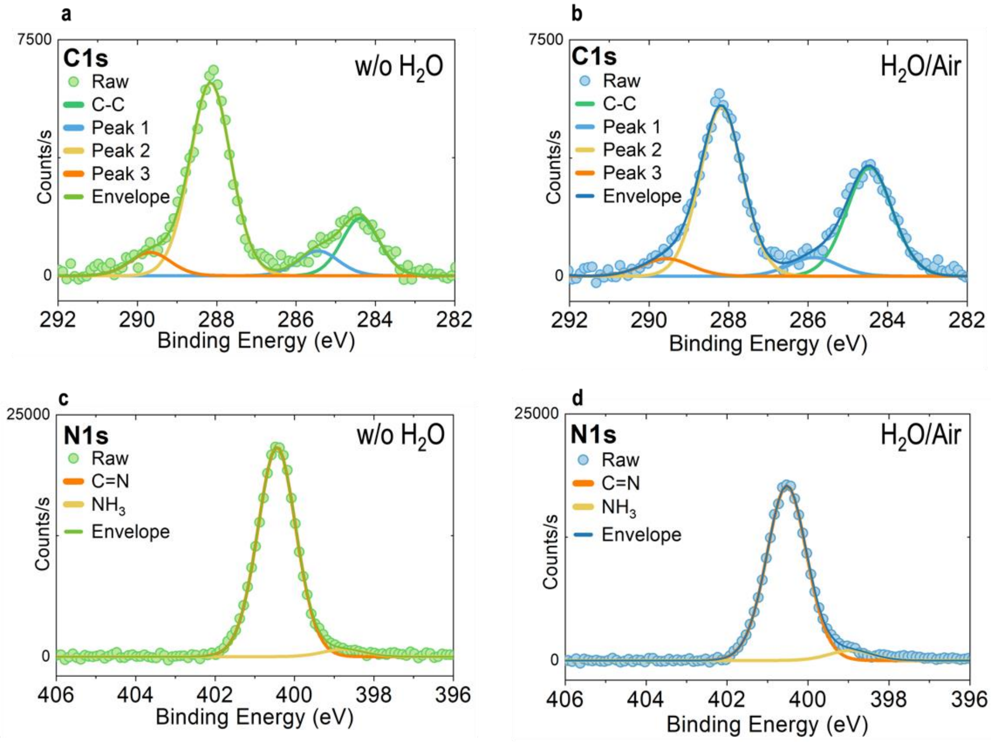

Figure S12. XPS spectra of $Cs_{0.17}FA_{0.83}PbI_3$ (CsFA) exposed *ex-situ* to H_2O/Air with relative humidity of 80% for 20 hours and peak deconvolution for **a)** C 1s w/o H_2O exposure, **b)** C 1s H_2O/Air exposure, **c)** N 1s w/o H_2O exposure, **d)** N 1s H_2O/Air exposure.

Table S2. XPS convoluted peaks details and atomic percent for $Cs_{0.17}FA_{0.83}PbI_3$ (CsFA) without (w/o) exposure to H_2O

CsFAPbI$_3$ –w/o H$_2$O					
	Name	BE eV	FWHM eV	Area (P) CPS.eV	Atomic %
**C 1s**	C-C	284.37	1.12	2221.37	2.84%
	Peak 1	285.44	1.21	1038.77	1.33%
	Peak 2	288.16	1.21	8075.49	10.18%
	Peak 3	289.68	1.21	978.11	1.25%
**N 1s**	C=N	400.44	1.14	26751.59	21.77%
	NH3	398.76	1.24	1030.11	0.83%
**O 1s**	Peak 1	533.1	3.37	2085.32	1.08%
**I 3d**	I3d5 Scan A	619.09	1.31	741127.39	47.79%
	I3d3 Scan A	630.56	1.31	513604.64	0.00%
**Pb 4f**	Pb4f7 Scan A	138.13	0.99	174414.28	10.51%
	Pb4f5 Scan A	143.03	0.99	137147.45	0.00%
**Cs 3d**	Cs3d5 Scan A	724.82	1.3	40130.21	2.42%
	Cs3d3 Scan A	738.75	1.31	27800.64	0.00%

Table S3. XPS convoluted peaks details and atomic percent for $Cs_{0.17}FA_{0.83}PbI_3$ exposed to H_2O/Air from Figure S11

CsFAPbI$_3$ - H$_2$O/Air					
	Name	BE eV	FWHM eV	Area (P) CPS.eV	Atomic %
C 1s	C-C	284.45	1.36	5096.07	5.71%
	Peak 1	285.86	1.46	936.78	1.05%
	Peak 2	288.18	1.26	7362.1	8.27%
	Peak 3	289.56	1.46	878.29	0.99%
N 1s	C=N	400.52	1.17	22404.59	16.19%
	NH3	398.94	1.26	1404.33	1.01%
O 1s	Peak 1	532.29	1.72	39460.16	18.30%
	Peak 2	530.37	1.82	3891.3	1.80%
I 3d	I3d5 Scan A	619.22	1.4	635074.14	36.38%
	I3d3 Scan A	630.68	1.41	440109.26	0.00%
Pb 4f	Pb4f7 Scan A	138.04	1.04	155980	8.49%
	Pb4f5 Scan A	142.94	1.04	122651.99	0.00%
Cs 3d	Cs3d5 Scan A	724.58	1.45	33320.34	1.79%
	Cs3d3 Scan A	738.52	1.45	23082.88	0.00%

Note on XPS measurements: XPS was performed under an ultra-high vacuum instrument. In addition, the samples were exposed for a few seconds between the transfer from a nitrogen glovebox to the measurement setup. We note that these measurements were done *ex-situ*, therefore, there was no possibility of measuring XPS to samples exposed to H_2O/N_2, nor only exposed to dry air. The *in-situ* setup was only for the GIWAXS measurements to study the structural phase transformations.

Fig. 11c shows the C1s peaks. The peak at ~284.5eV from the C−C and C−H bonds. Two minor peaks resulted from the peak deconvolution (**Fig. S12a-b**) that may be attributed to other C-N complexes.[14–16] The peak at ~288.16eV, peak 2, is principally attributed to C=N bond from FA. After H_2O/Air exposure, Peak 1, corresponding to C−C, C−H, or C−N bonds increases intensity, indicating other carbon species forming at the surface, such as carbonates, possibly from atmospheric contamination (**Fig. S12**). After H_2O/Air exposure, Peak 2, the C=N peak intensity decreases, in line with the FA^+ loss. However, the atomic percentage attributed to Peak 2 decreases only 2% compared to the 5.6 % decrease in from the nitrogen from the N1s spectra (**Tables S2-3**). For this reason, we also attribute Peak 2 from the C1s spectra in **Fig. S12b,** after H_2O/Air exposure, to Carbon-Oxygen complexes such as carbonates or carboxyls, that have a binding energy from 288 to 291eV.[16]

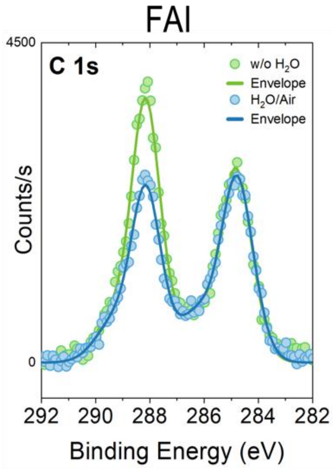

Figure S13. XPS of FAI thin films deposited in a nitrogen glovebox and exposed for few minutes to air before in the transfer to XPS instrument w/o H_2O exposure or exposed to H_2O/Air with a relative humidity of 80% for 20 hours. Spectra of the C 1s.

Table S4. XPS convoluted peaks details and atomic percent for FAI films without (w/o) H_2O exposure

FAI – w/o H_2O					
	**Name**	**BE eV**	**FWHM eV**	**Area (P) CPS.eV**	**Atomic %**
**C 1s**	C-C	284.83	1.39	4073.14	5.79
	Peak 1	286.41	1.49	957.17	1.36
	Peak 2	288.16	1.29	5146.65	7.33
	Peak 3	289.53	1.29	745.49	1.06
**N 1s**	C=N	400.37	1.4	17927.23	16.42
**O 1s**	Peak 1	532.78	1.62	94305.83	55.47
	Peak 2	530.8	1.59	6906.38	4.06
**I 3d**	I3d5 Scan A	618.8	1.49	117297.44	8.51
	I3d3 Scan A	630.26	1.49	81287.66	0

Table S5. XPS convoluted peaks details and atomic percent for FAI thin films exposed to H_2O/Air

FAI - H_2O/Air					
	Name	BE eV	FWHM eV	Area (P) CPS.eV	Atomic %
C 1s	C-C	284.79	1.43	3997.97	5.36
	Peak 1	286.33	1.52	1017.77	1.37
	Peak 2	288.15	1.33	3480.2	4.67
	Peak 3	289.47	1.52	770.96	1.04
N 1s	C=N	400.38	1.44	12185	10.53
O 1s	Peak 1	532.8	1.62	122020.69	67.71
	Peak 2	530.8	1.6	7659.7	4.24
I 3d	I3d5 Scan A	618.83	1.47	74209.34	5.08
	I3d3 Scan A	630.29	1.47	51427.41	0

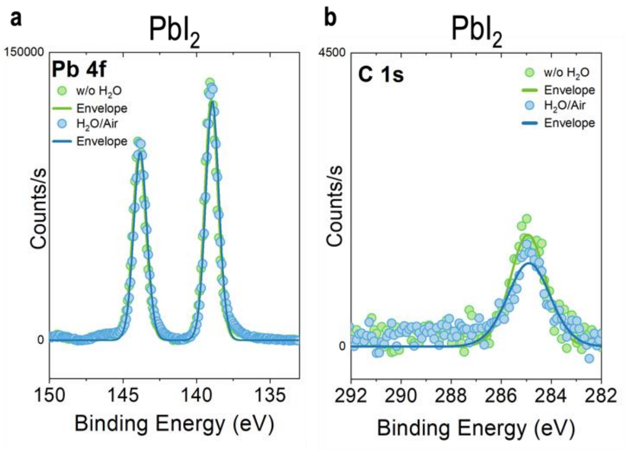

Figure S14. XPS of PbI_2 thin films deposited in a nitrogen glovebox and exposed for few minutes to air before in the transfer to XPS instrument w/o H_2O exposure or exposed to H_2O/Air with a relative humidity of 80% for 20 hours. **a)** Pb 4f, **b)** C 1s.

Table S6. XPS convoluted peaks details and atomic percent for PbI_2 films without (w/o) H_2O exposure

PbI_2 –w/o H_2O					
	Name	BE eV	FWHM eV	Area (P) CPS.eV	Atomic %
C 1s	C-C	284.93	1.68	3119.31	11.58
I 3d	I3d5 Scan A	619.9	1.26	331536.1	62.95
	I3d3 Scan A	631.36	1.26	229756.02	0
Pb 4f	Pb4f7 Scan A	139.01	1.05	143537.9	25.47
	Pb4f5 Scan A	143.91	1.05	112868.37	0

Table S7. XPS convoluted peaks details and atomic percent for PbI_2 thin films exposed to H_2O/Air

PbI_2 - H_2O/Air					
	Name	BE eV	FWHM eV	Area (P) CPS.eV	Atomic %
C 1s	C-C	284.89	2.01	2789.37	10.58
I 3d	I3d5 Scan A	619.75	1.24	327731.4	63.57
	I3d3 Scan A	631.21	1.25	227119.34	0
Pb 4f	Pb4f7 Scan A	138.92	1.05	142544.16	25.84
	Pb4f5 Scan A	143.82	1.05	112086.96	0

2.2 Bulk chemistry

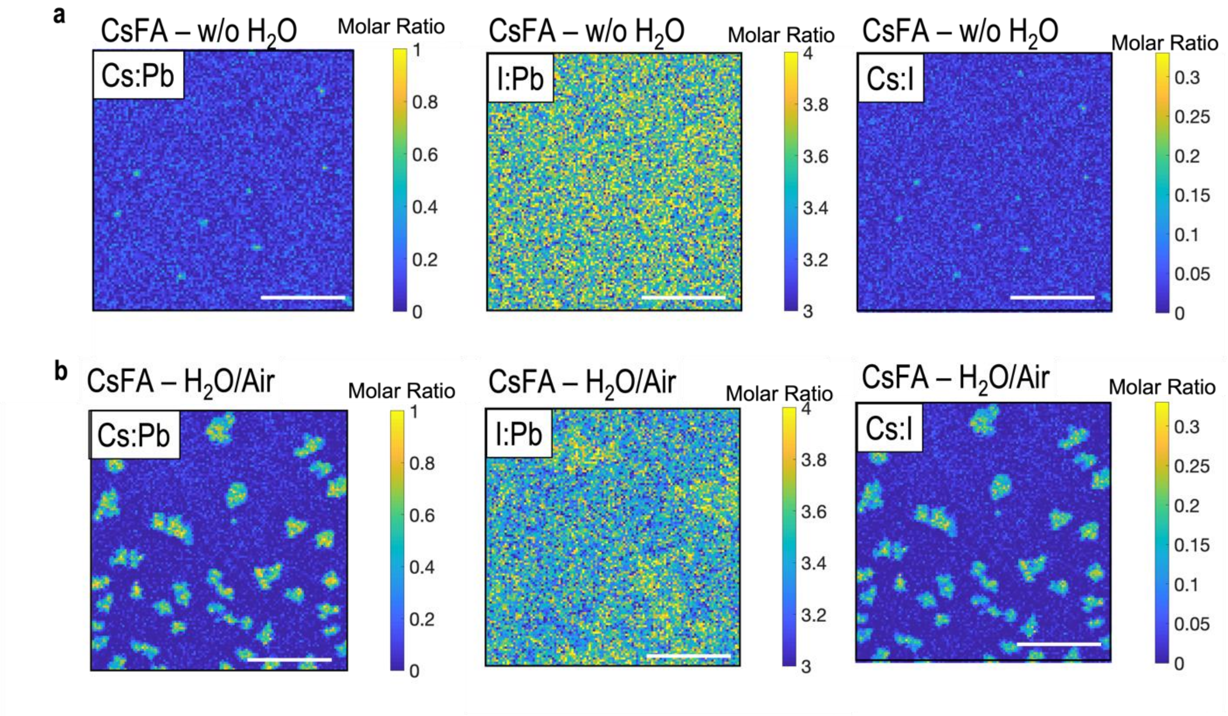

Figure S15. X-ray fluorescence (XRF) maps of the molar ratios between Cs, Pb, and I in $Cs_{0.17}FA_{0.83}PbI_3$ (CsFA) thin films **a)** without (w/o) H_2O exposure. The samples were exposed to air before the measurement in a helium-vented chamber. **b)** after *ex-situ* exposure to H_2O/Air with relative humidity 80% for 20 hours. The measurement was in a helium-vented chamber. The scale bar is 10μm.

Note on XRF: XRF measurements were done to CsFA films on glass to prevent fluorescence signal from the ITO. It is important to highlight that the characteristic X-ray fluorescence emission energies of Cs and I are close in energy and overlap. For this reason, even if we quantify the mass and molar values with a standard, there is loss of accuracy due to the low and overlapped energies.

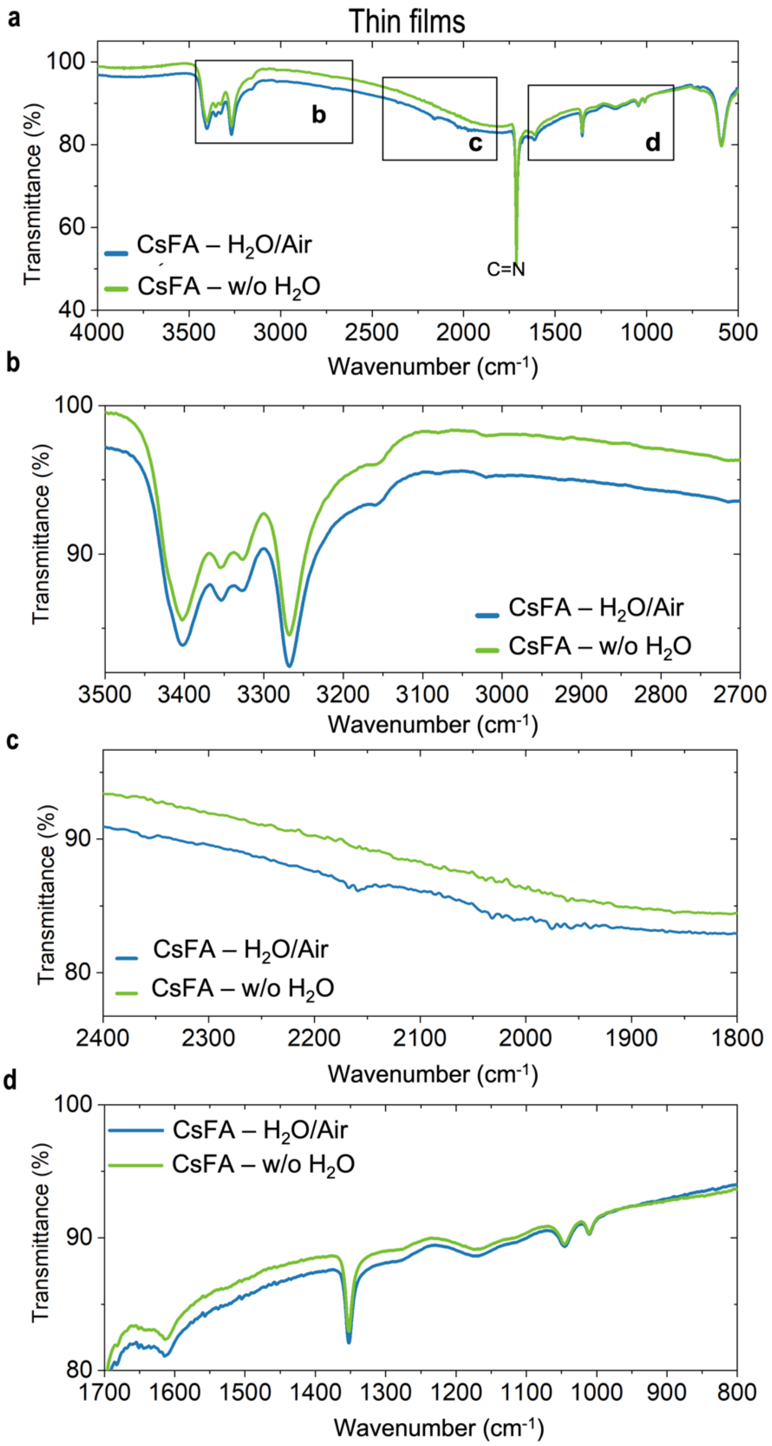

Figure S16. FTIR $Cs_{0.17}FA_{0.83}PbI_3$ thin films (CsFA). (green) Without (w/o) H_2O exposure deposited, and synthesized in a nitrogen glovebox, and measured in air. (blue) CsFA films after *ex-situ* exposure to H_2O/Air with relative humidity 80% for 20 hours

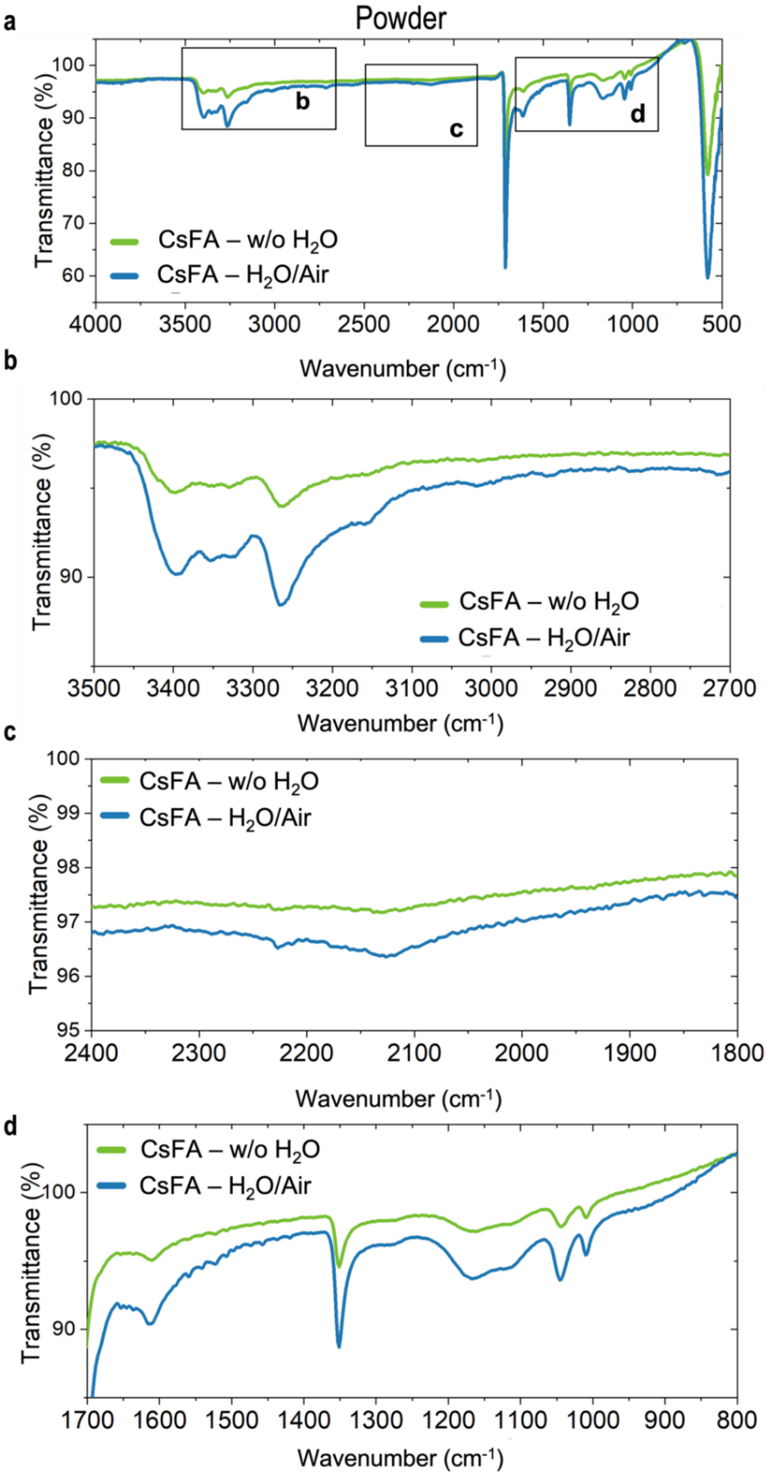

Figure S17. FTIR $Cs_{0.17}FA_{0.83}PbI_3$ powders (CsFA). (green) Without (w/o) H_2O exposure, deposited, and synthesized in a nitrogen glovebox, and measured in nitrogen glovebox without air exposure. (blue) CsFA films after *ex-situ* exposure to H_2O/Air with relative humidity 80% for 20 hours.

Table S8. FTIR peaks and assignment for powders and thin films [17–20]

Wavenumber (cm^{-1})		
Powder	Thin Films	Peak Assignment
3400	3403	N-H stretches
3352	3353	
3324	3325	
3264	3267	Symmetric NH_3^+ stretch
3156	3156	Asymmetric NH_3^+ stretch
1708	1705	C=N stretch Symmetric
1600	1610	Skeletal C-C, C-N bending
1350	1352	
1275	1270	
1167	1172	
1043	1047	
1007	1010	
579	595.9	

2.4 DFT calculations

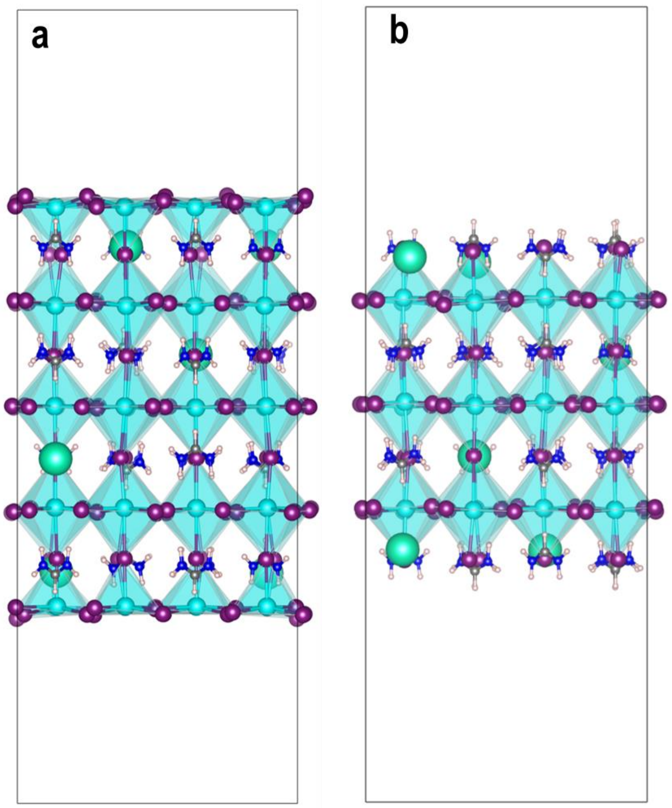

Figure S18. Surface models of $Cs_{0.2}FA_{0.8}PbI_3$ with **a)** PbI_2-termination and **b)** CsFAI-termination. Following color code is used for the atomic representations: purple, I; cyan, Pb; blue, N; green, Cs; gray, C; white, H.

Table S9. Adsorption energy of O_2 on PbI_2- and CsFAI-terminated perovskite surfaces, see Figure S17. Values in parentheses are given without D3 corrections.
[a] The hydrated PbI_2-terminated slab was used as reference to calculate the O_2 adsorption energy.

Surface	Adsorption Site	Adsorption energy / eV
PbI_2-terminated	Pb	-0.17 (-0.05)
	Cs top site	-0.22 (-0.05)
	FA top site	-0.03 (-0.04)
	V_I	-0.21 (-0.09)
	Hydrated [a]	-0.31 (0.07)
CsFAI-terminated	Cs top site	0.34 (0.27)
	FA top site	0.06 (0.11)

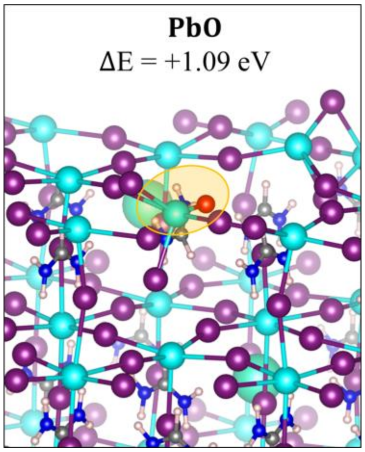

Figure S19. DFT calculation of PbO formation at a defective PbI_2-terminated surface. An oxygen atom is placed at an iodide vacancy and bonded to Pb as initial setup. The relaxed geometry shows a reaction energy of +1.09 eV, being thermodynamically unfavorable.

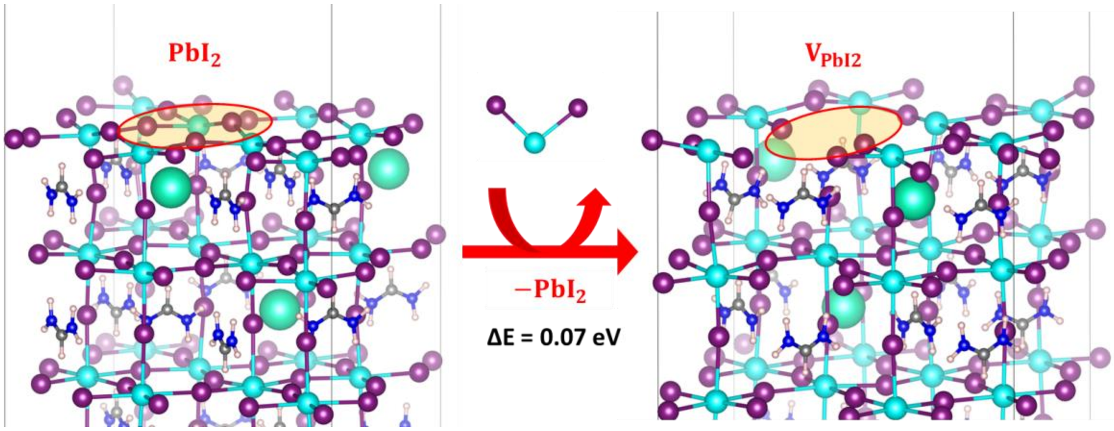

Figure S20. DFT calculation of PbI_2 vacancy formation: (left) pristine PbI_2-terminated surface, (right) defective surface containing a PbI_2 vacancy. The reaction occurs by removal of a PbI_2 unit from the surface, showing positive reaction energy of 0.07 eV. Following color code is used for the atomic representations: purple, I; cyan, Pb; blue, N; green, Cs; gray, C; white, H.

Table S10. Energy of π* orbitals of molecular oxygen in vacuum and hydrated on the B3LYP/6-311G** level of theory with empirical D3 corrections. Effect of water is modeled by placing 4 water molecules next to the oxygen and the use of an implicit solvation model in Gaussian09 (see computational details).

Environment	Orbital energy / eV
Vacuum	-3.185
Water	-3.439/-3.468

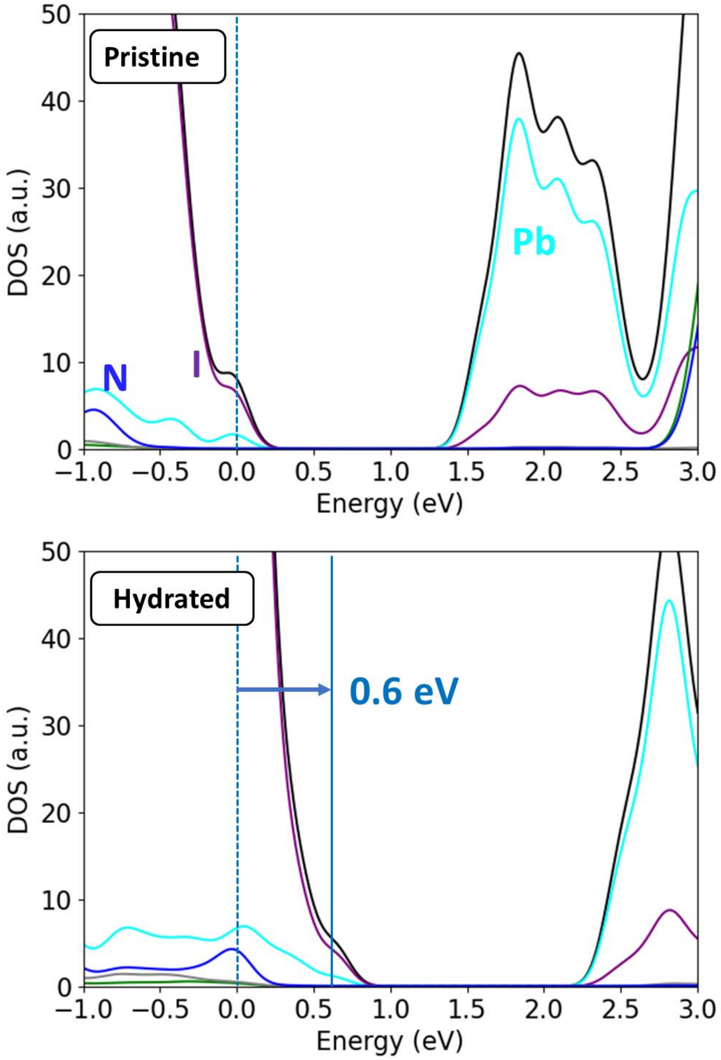

Figure S21. Density of states (DOS) of the perovskite slab for the pristine and hydrated surface. All DOS are aligned at the C 2s peak of a bulk FA cation, and energies are referenced to the VBM of the pristine surface. The shift in VBM is explicitly highlighted in the lower panel. Following color code is used for the atomic representations: purple, I; cyan, Pb; blue, N; green, Cs; gray, C; white, H.

S2.5 DFT calculations of superoxide formation from light and from mobile ions

Hypothesis:

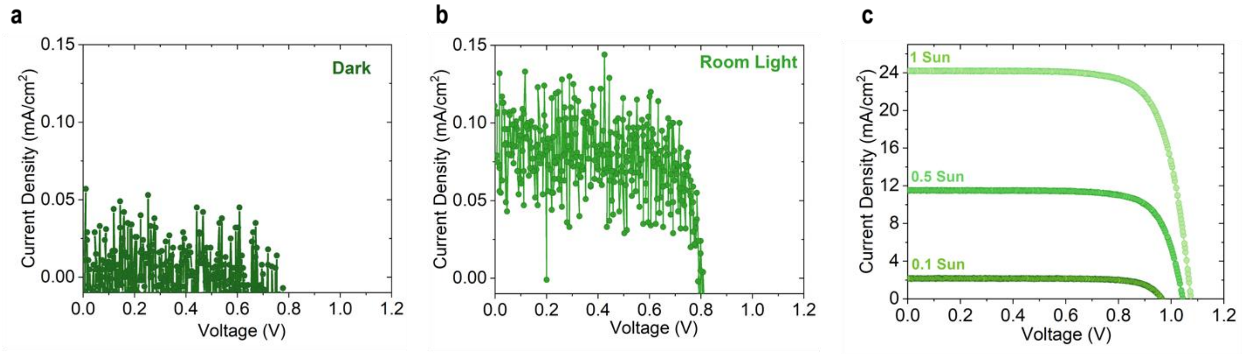

Figure S22. Current-voltage curves of a $Cs_{0.17}FA_{0.83}PbI_3$ solar cell of the architecture shown in **Fig.5d** of the main text, untreated. **a)** Dark, **b)** room light of the laboratory where the measurement was done, non-quantified, **c)** comparison of different sun-light intensities: 0.1 sun, 0.5 sun, and 1 sun with intensity AM 1.5.

The current-voltage characteristic of a CsFA perovskite solar cell at room light shows how for a low-intensity, not quantified, room light the device creates a photocurrent and photovoltage. Free charge carriers can be generated from a regular source of light. In addition, we show in **Fig. S22c** how the current density and voltage changes as a function of light-intensity, quantified by 1 sun of illumination AM1.5.

We performed DFT calculations of superoxide formation in H_2O/Air and from mobile ions. Recent studies raised the importance of superoxide in perovskite degradation from photooxidation.[21–24] Here, we consider potential superoxide ($O_2^{\bullet-}$) and peroxide (O_2^{2-}) formation mechanisms from mobile iodine ions (*Rx. S1a* and *S1b*) and from light (*Rx. S2*):

$$2I^- + 2O_2 \rightarrow 2O_2^{\cdot-} + I_{2(g)} \quad [S1a]$$

$$2I^- + O_2 \rightarrow O_2^{2-} + I_{2(g)} \quad [S1b]$$

$$O_2 + h\nu \longrightarrow O_2 + e^- + h^+ \longrightarrow O_2^{\cdot-} + h^+ \quad [S2]$$

Reaction S1 is considered at the FAI-terminated surface, where oxygen molecules are adsorbed on iodide vacancies, forming molecular iodine I_2 by superoxide or peroxide formation, see **Fig. S23**. DFT calculations predict a formation energy of +0.81 eV for reaction 1a, being thermodynamically unfavorable in dark. For the formation of peroxide, we obtain a reduced formation energy of +0.65 eV, while the optimized structure shows a deprotonated FA cation by peroxide, resulting in HOO^-. However, both processes are thermodynamically unfavorable, suggesting a reduced relevance of iodine oxidation via superoxide/peroxide formation.

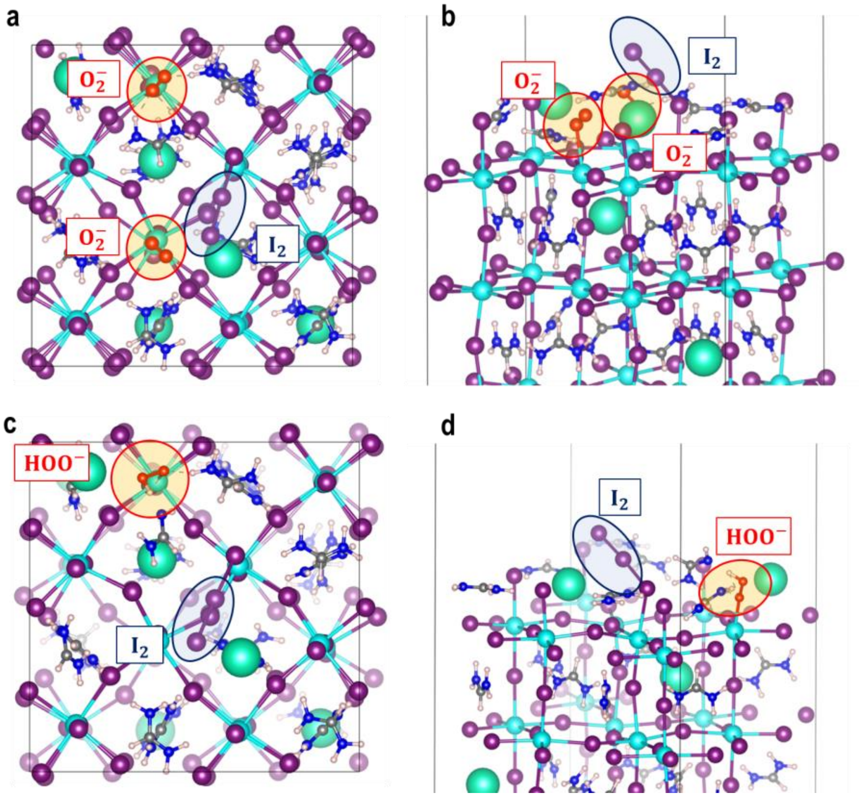

Figure S23. Top and side view of the FAI-terminated perovskite surface with oxidized iodine, I_2, via (**a,b**) superoxide [reaction 1a] and (**c,d**) peroxide formation [reaction 1b]. Following color code is used for the atomic representations: purple, I; cyan, Pb; blue, N; green, Cs; gray, C; white, H.

Reaction S2 starts from O_2 adsorption on the surface, followed by the photoexcitation of the perovskite at light, and subsequent electron transfer from the perovskite to the O_2, resulting in $O_2^{\bullet-}$ as shown in **Fig. S24**. The superoxide formation energy from reaction [S2] is modeled in line with literature [24] using the following equation:

$$E_{react}([2]) = E(slab + O_2^{\bullet-}) - E(O_2) - E(slab^{-}) + E_{gap}$$

where $E(slab + O_2^{\bullet-})$ is the total energy of the CsFA perovskite slab containing the adsorbed superoxide $O_2^{\bullet-}$; $E(O_2)$ is the energy of an isolated O_2 molecule; $E(slab^{-})$ is the energy of the CsFA perovskite slab in excited state with charge -1; E_{gap} is the band gap of the CsFA perovskite to generate the charge carriers.

Regular room light creates a small photo-current and photo-voltage in a CsFA perovskite solar cell (**Fig. S22**), thus reaction S2 is more likely in explicit illumination at 1 sun intensity. We explicitly consider *reaction S2* at different $Cs_{0.2}FA_{0.8}PbI_3$ surfaces. Considering the electronic structure of the pristine surface in **Fig. S25**, the unoccupied π^* orbital of the adsorbed O_2 is close to the conduction band of the perovskite surface. On the hydrated surface in **Fig. S25b**, H_2O molecules enhance O_2 adsorption, E_{ads} = -0.31 eV, and introduce a striking shift of the π^* orbital from 1.44 eV to 0.44 eV above the valence band maximum (VBM) as seen the hydrated one. H_2O molecules strongly bond with undercoordinated surface Pb ions, resulting in an upshift of VBM by 0.58 eV. Moreover, the enhanced O_2 attraction yields a stabilization of the π^* orbital by 0.42 eV.

Interestingly, O_2 adsorbed at defects such as V_I^+ (E_{ads} = -0.21 eV) results in a comparable shift of the unoccupied π* orbital with respect to the VBM, mainly due to the downshift of the π* orbital by 0.72 eV (Defect in **Figs. S25a** and **b**).

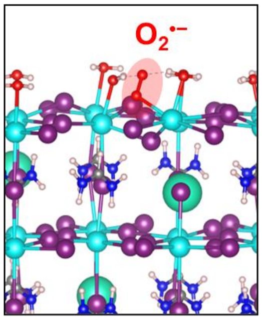

Figure S24. Light induced superoxide at the hydrated PbI_2-terminated perovskite surface [reaction 2]. Following color code is used for the atomic representations: purple, I; cyan, Pb; blue, N; green, Cs; gray, C; white, H.

Finally, we computed the superoxide formation energies from the above mechanisms. The formation of superoxide by I_2 release, *reaction S1*, shows a large energy of 0.81 eV, higher than the energies of reaction S2 seen in **Fig. S26**. Upon illumination, we observe similar energies for $O_2^{\bullet-}$ formation at the pristine and at the defective surfaces. In contrast, we obtain a substantial reduction in $O_2^{\bullet-}$ formation energy to 0.39 eV at the hydrated surface, which can be attributed to the stabilization of $O_2^{\bullet-}$ by hydrogen bonding with H_2O molecules. Consequently, the low formation energy suggests an increased superoxide yield at the hydrated CsFA perovskite surface compared to the pristine dry surface under light illumination.

We hypothesize on subsequent reactions driven by superoxide in a H_2O/Air atmosphere. $O_2^{\bullet-}$ reacts with adsorbed water molecules to form hydroperoxyl, HO_2^-, and hydroxyl radicals, OH^- [*Rx.S3*].[25] These oxygen species catalyze further reactions with water, forming hydrogen peroxide, H_2O_2, among other products [*Rx. S4*].[23,26] Reactive species, such as OH^-, rapidly deprotonate the FA^+ [*Rx. S5*].

$$2O_2^{\cdot-} + H_2O \longrightarrow O_2 + HO_2^- + OH^- \; [S3]$$
$$HO_2^- + H_2O \longrightarrow OH^- + H_2O_2 \; [S4]$$
$$OH^- + FA^+ \rightarrow H_2O + FA_{(g)} \; [S5]$$

In addition to the chemical reactions described in the text, we consider other reactive species in the air such as CO_2, we show a possible chemical reaction of CO_2 with water [*Rx. S6*] that leads to the formation of carbonates. Other reactions associated with Pb species are shown in [*Rx. S6 - S8*].

$$CO_2 + H_2O \longrightarrow CO_3^{-2} + 2H^+ \; [S6]$$
$$PbI_2 + 2H_2O \rightarrow PbOH_2 + 2HI \; [S7]$$
$$Pb^{+2} + CO_3^{-2} + Pb + H_2O_2 \longrightarrow PbCO_3 + PbO \; [S8]$$

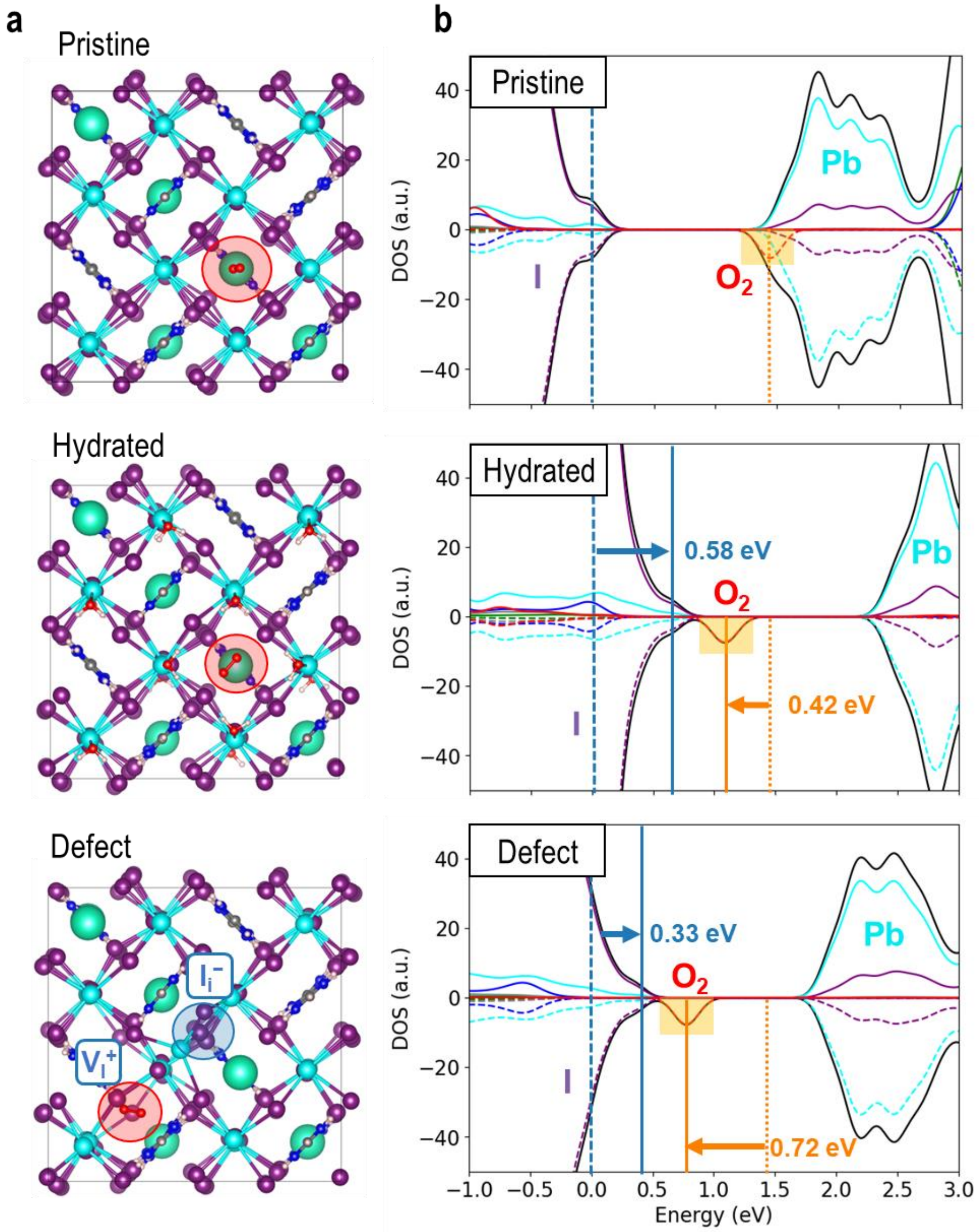

Figure S25. **a)** Top view of the PbI_2-terminated perovskite surface after O_2 adsorption on the (top) pristine, (middle) hydrated, and (bottom) defective surface on an iodide vacancy site. The adsorbed O_2 molecule is highlight by a red circle. **b)** Density of states (DOS) from spin-polarized DFT calculations for the pristine, hydrated, and defective surfaces after O_2 adsorption. The unoccupied π^* orbitals of the O_2 molecule are explicitly highlighted. All DOS are aligned at the C 2s peak of a bulk FA cation, and energies are referenced to the VBM of the pristine surface.

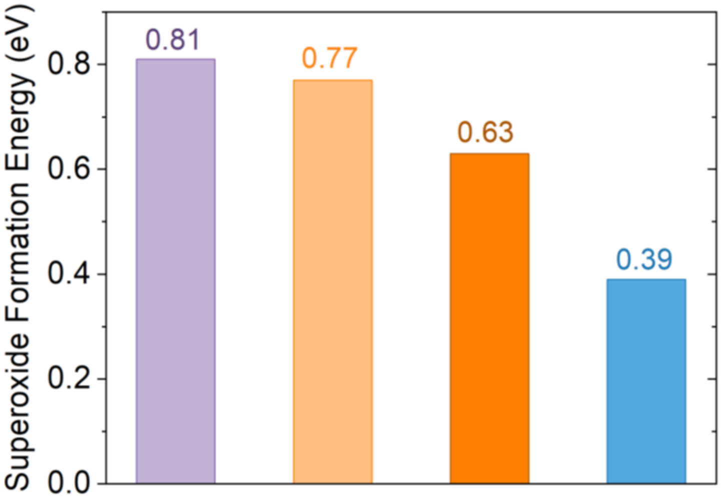

Figure S26. Superoxide formation energies from mobile ions [*Rx.* S1], and from light irradiation of the pristine, defective, and hydrated CsFA perovskite surface [*Rx.* S2].

2.6 Stabilizing the perovskite phase

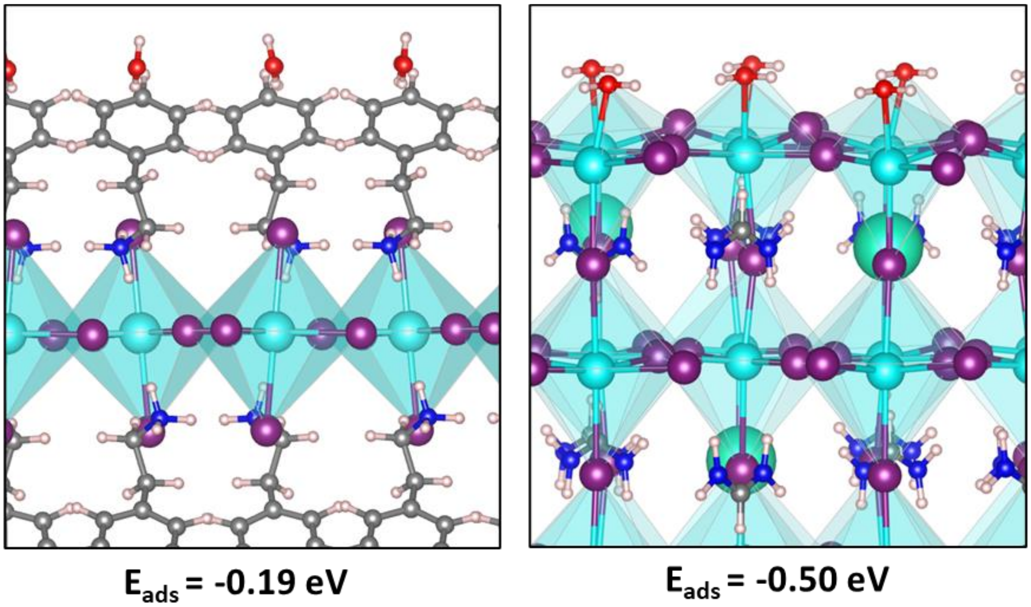

Figure S27. Water molecule adsorption on the (left) PEAI-terminated surface of a two-dimensional PEA_2PbI_4 perovskite, and (right) PbI_2-terminated surface of the considered CsFA perovskite. Adsorption energies are given, normalized per water molecule.

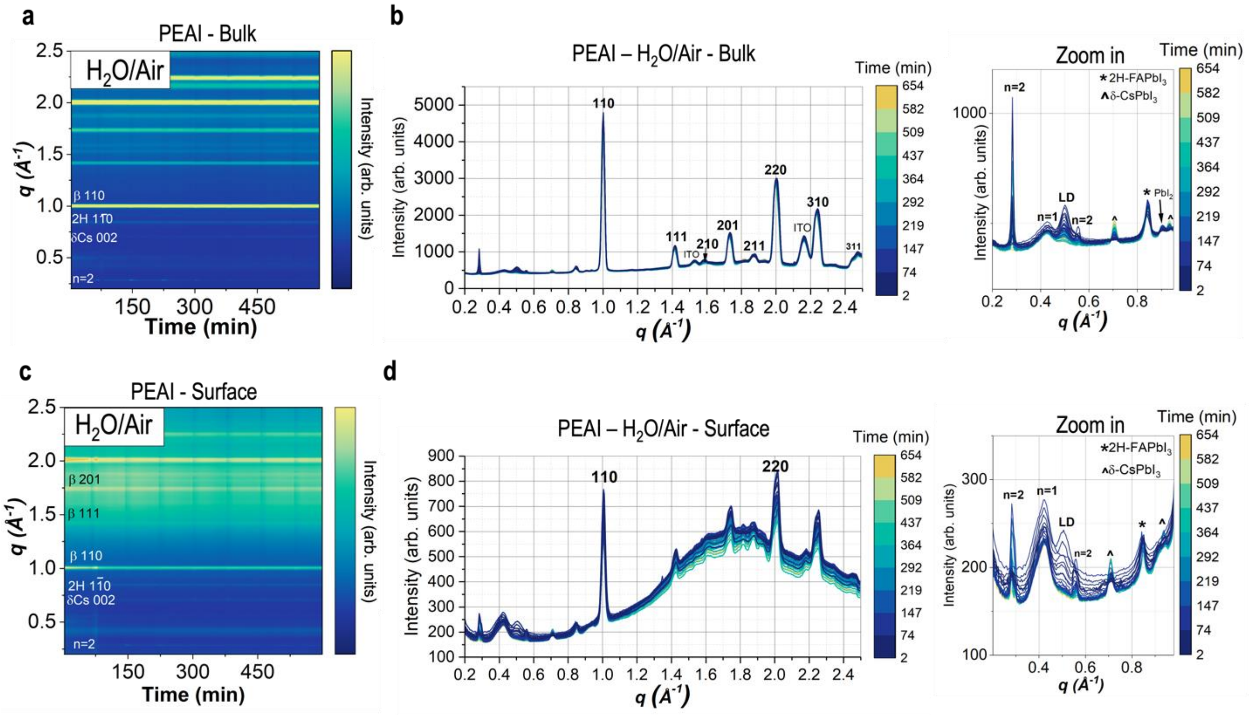

Figure S28. *In-situ* GIWAXS in H_2O/Air of PEAI-treated samples. Bulk (incident angle 0.5°) **a)** scattering vector as a function of time exposed to H_2O/Air bulk, **b)** integrated circular average of the diffraction at the bulk (incident angle 0.5°) and zoom in the PEAI and low-dimensional (LD) phases region bulk. Surface (incident angle 0.1°) c) scattering vector as a function of time exposed to H2O/Air surface, d) integrated circular average of the diffraction at the surface (incident angle 0.1°) and zoom in the PEAI and low-dimensional (LD) phases region surface.

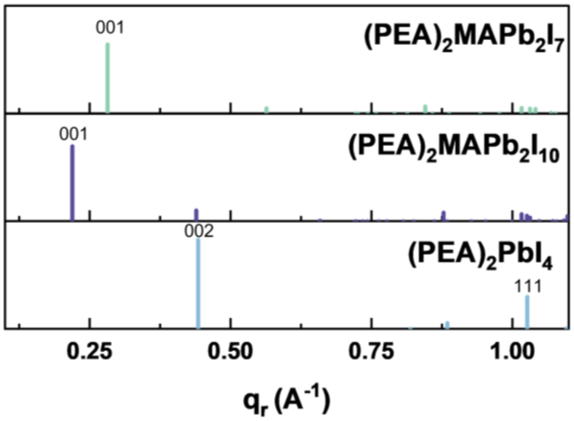

Figure S29. Simulated diffraction files for low dimensional (LD) PEA cation Ruddlesden Popper phases.

Note on in-situ GIWAXS with PEAI: We observe differences between the surface and bulk measurements. At the surface, there is more n=1 Ruddlesden popper phase (PEA_2PbI_4), while at the bulk it converts into n=2 or other LD PEA-phases.

We analyzed the evolution of the main peaks following the procedure done for **Figs. S3** and **S4**, but in this case for the PEAI-treated devices exposed to H_2O/Air in **Fig. S30**. We point out that the non-perovskite phase 2H decreases as a function of time exposed to H_2O/Air. This may be

correlated to another mechanism occurring in parallel to the exposure to H_2O and O_2, of the reaction of the 2H phase with PEAI to form a LD Ruddlsden-Popper phase.

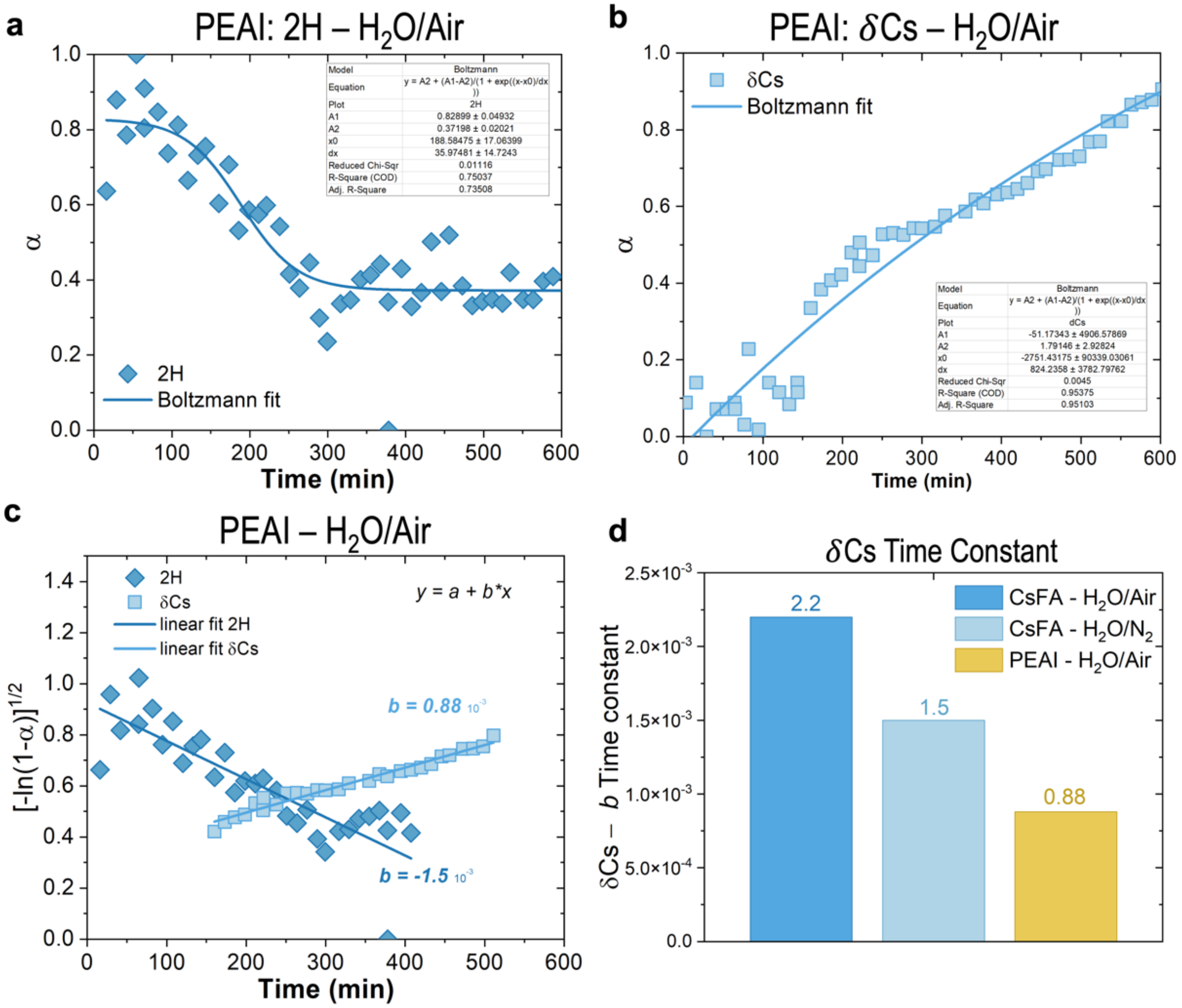

Model	Boltzmann
Equation	y = A2 + (A1-A2)/(1 + exp((x-x0)/dx))
Plot	2H
A1	0.82899 ± 0.04932
A2	0.37198 ± 0.02021
x0	188.58475 ± 17.06399
dx	35.97481 ± 14.7243
Reduced Chi-Sqr	0.01116
R-Square (COD)	0.75037
Adj. R-Square	0.73508

Model	Boltzmann
Equation	y = A2 + (A1-A2)/(1 + exp((x-x0)/dx))
Plot	dCs
A1	-51.17343 ± 4906.57869
A2	1.79146 ± 2.92824
x0	-2751.43175 ± 90339.03061
dx	824.2358 ± 3782.79762
Reduced Chi-Sqr	0.0045
R-Square (COD)	0.95375
Adj. R-Square	0.95103

PEAI: 2H – H_2O/Air - linear fit

Equation	y = a + b*x
Plot	2h log
Weight	No Weighting
Intercept	0.92557 ± 0.0416
Slope	-0.0015 ± 1.68649E-4
Residual Sum of Squares	0.37944
Pearson's r	-0.85075
R-Square (COD)	0.72377
Adj. R-Square	0.71456

PEAI: δCs – H_2O/Air - linear fit

Equation	y = a + b*x
Plot	dCslog
Weight	No Weighting
Intercept	0.31913 ± 0.01067
Slope	8.8152E-4 ± 3.06916E-5
Residual Sum of Squares	0.00788
Pearson's r	0.9846
R-Square (COD)	0.96945
Adj. R-Square	0.96827

Figure S30. Peak evolution from integrated area normalized into α and fitted to a Boltzmann sigmoidal function **a)** PEAI-treated 2H peak in H_2O/Air, **b)** PEAI-treated δCs peak in H_2O/Air. Data fitted into a linear logarithmic function to compare the evolution and calculate time constant (b) for **c)** PEAI-treated films in H_2O/Air.

2.6.1 Solar cells with PEAI

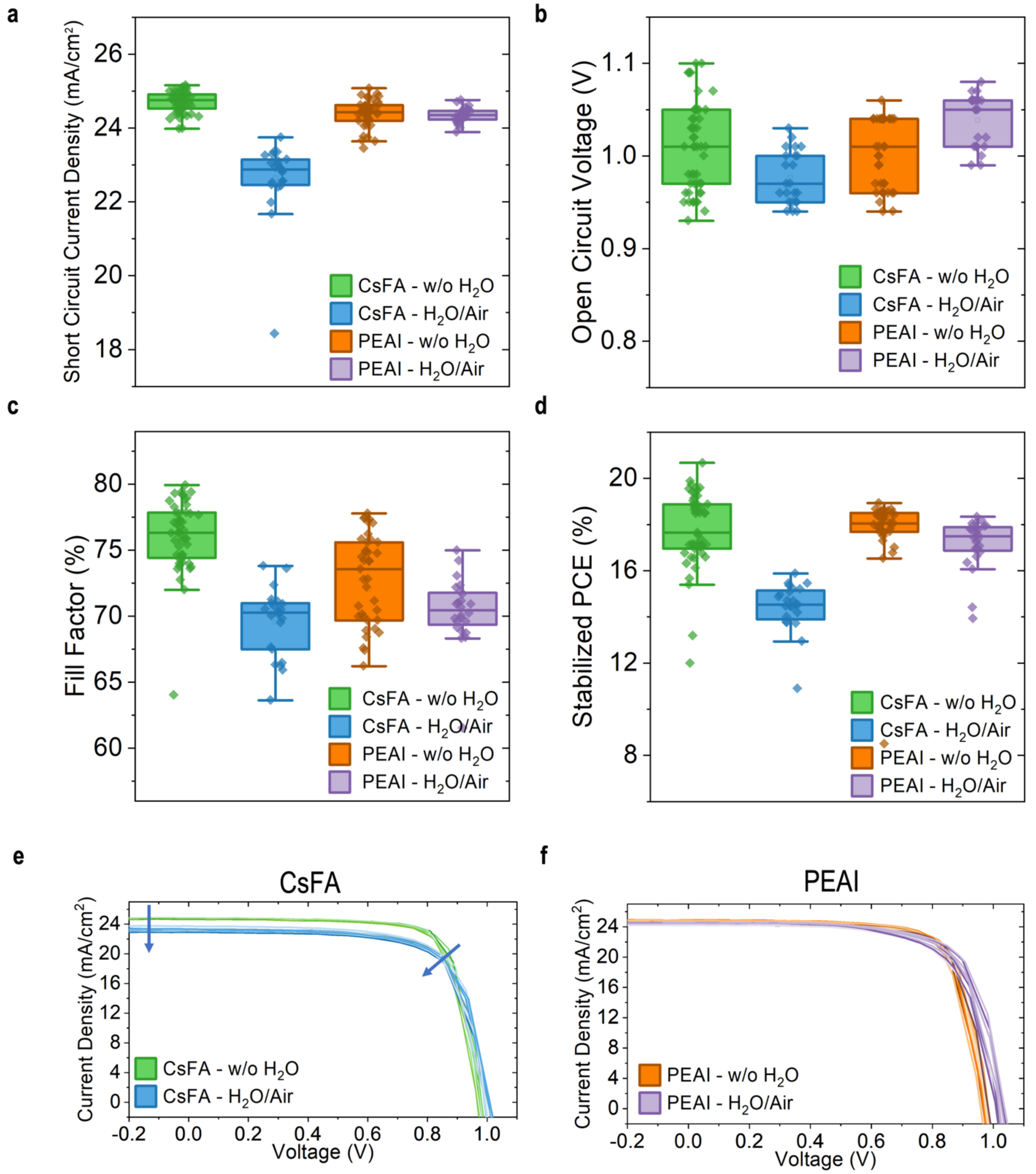

Figure S31. Solar cells result for the untreated CsFA perovskites, without (w/o) H_2O exposure, after CsFA is *ex-situ* exposed to H_2O/Air, and for PEAI-treated w/o H_2O exposure and after *ex-situ* H_2O/Air exposure. H_2O exposure is done before the Sprio-OMeTAD and Au layers. Figures of merit show: **a)** Short-circuit current density, **b)** open circuit voltage, **c)** fill factor, **d)** stabilized power conversion efficiency (PCE). Example of current density- voltage curves for **e)** untreated CsFA and **f)** PEAI-treated devices without abd with H_2O/Air exposure.

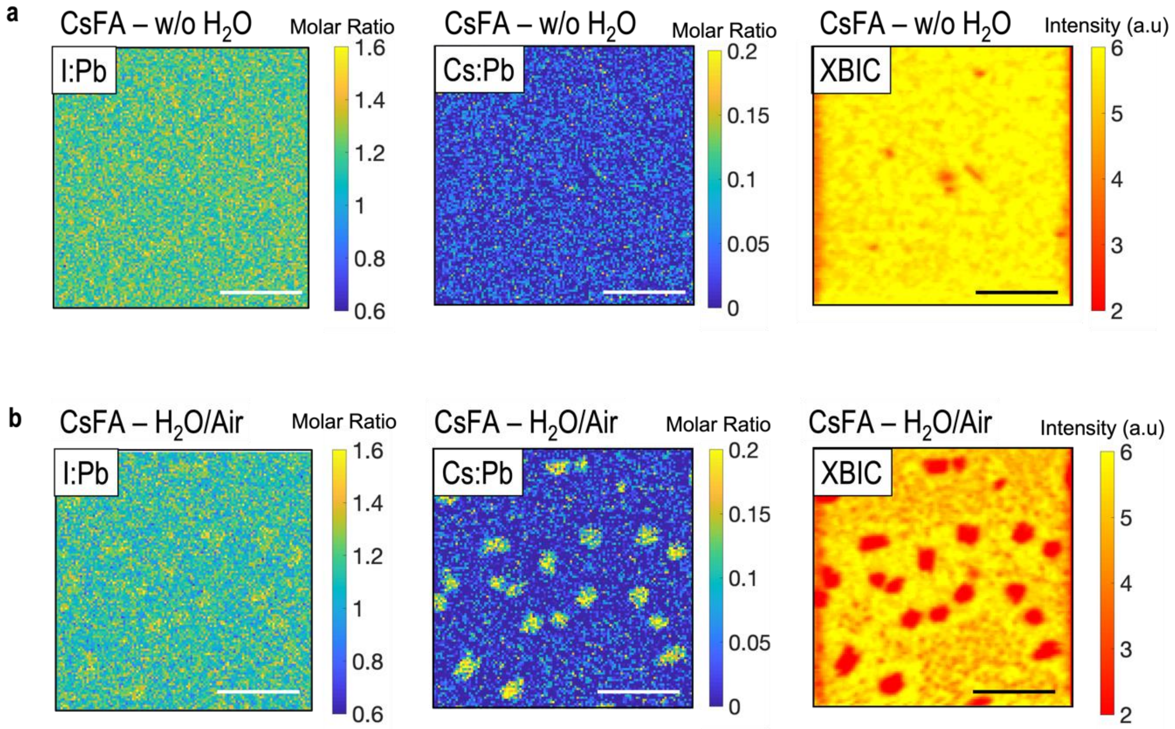

Figure S32. X-ray fluorescence (XRF) with correlative X-ray beam-induced current (XBIC) done on complete $Cs_{0.17}FA_{0.83}PbI_3$ solar cell devices. **a)** Without (w/o) H_2O exposure. The samples were exposed to air before the measurement, and the experiment was done in a helium-vented chamber. and **b)** after *ex-situ* exposure to H_2O/Air with relative humidity 80% for 20 hours. The measurements were done in a Helium-vented chamber. (I:Pb) denotes the calculated molar ratio iodine to lead from mass per area data. (Cs:Pb) denotes the molar ratio cesium to lead. The scale bar is 10μm.

XRF with correlative XBIC was performed to complete perovskite solar cells (see schematic in **Fig. 5** of the main text). The exposure to H_2O was done *ex-situ* during fabrication, as specified on the additional online methods. From the XRF measurements we obtain and quantify a mass per area value, which we later convert to a molar value to compare the molar ratio between I:Pb and Cs:Pb. It is important to consider that these values are comparable but not quantitatively accurate, given that the top layers of Spiro-OMeTAD, and Au, will affect the fluorescence signal. The main result from XRF-XBIC is the appearance of Cs-rich clusters that are correlated with a decrease in induced current. We also observe a relative decrease in I:Pb ratio after H_2O/Air exposure, in line with the XRF results in **Fig. S15**.

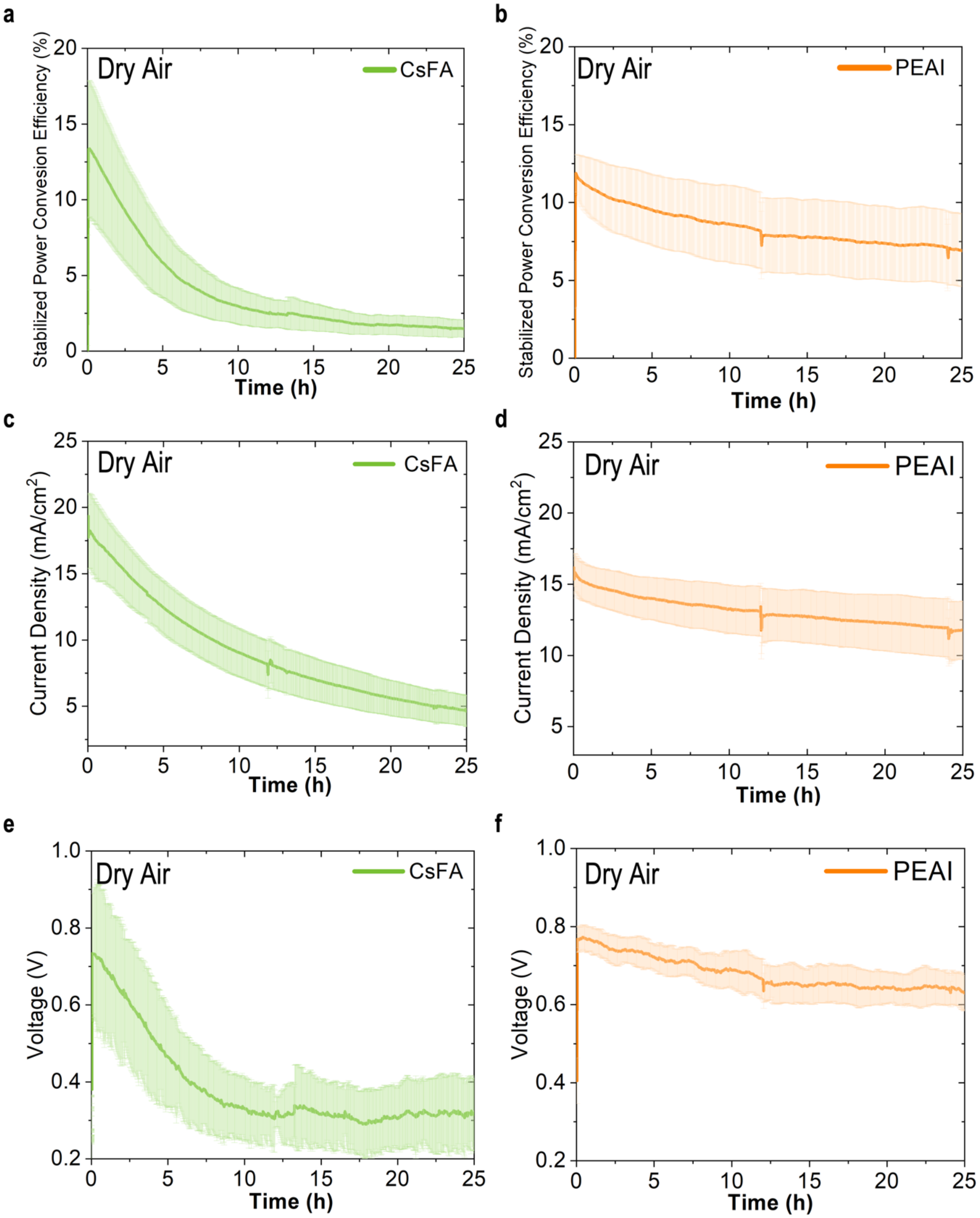

Figure S33. Long-term stability of the solar cells of $Cs_{0.17}FA_{0.83}PbI_3$ without and with a top layer of PEAI stressed constantly over one-sun illumination in dry air. The results show the average (bold line) of 16 devices with an area of 0.128 cm^2, and the standard deviation (light line). **a,b)** Stabilized power conversion efficiency (%) as a function of time in **a)** CsFA perovskite solar cell, and **b)** with PEAI-treated. **c,d)** Current-density as a function of time in **c)** CsFA perovskite solar cell, **d)** PEAI-treated. **e,f)** Voltage as a function of time in a **e)** CsFA perovskite solar cell, and **f)** PEAI-treated.

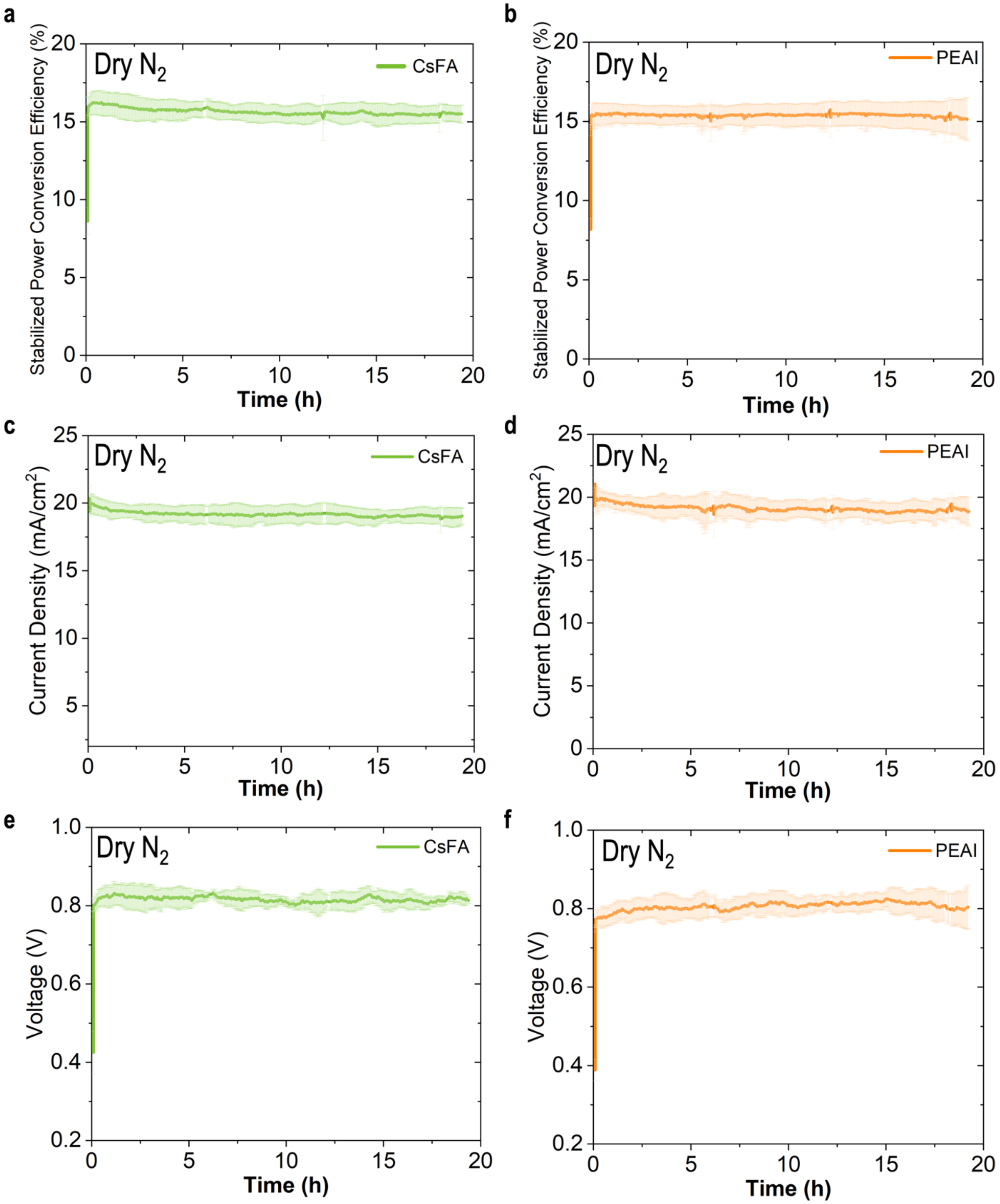

Figure S34. Long-term stability of the solar cells of $Cs_{0.17}FA_{0.83}PbI_3$ without and with a top layer of PEAI stressed constantly over one-sun illumination in dry nitrogen (N_2). The results show the average (bold line) of 8 devices with an area of 0.128 cm^2, and the standard deviation (light line). **a,b)** Stabilized power conversion efficiency (%) as a function of time in **a)** CsFA perovskite solar cell, and **b)** with PEAI-treated. **c,d)** Current-density as a function of time in **c)** CsFA perovskite solar cell, **d)** PEAI-treated. **e,f)** Voltage as a function of time in a **e)** CsFA perovskite solar cell, and **f)** PEAI-treated

3. References

(1) Saliba, M.; Correa-Baena, J. P.; Wolff, C. M.; Stolterfoht, M.; Phung, N.; Albrecht, S.; Neher, D.; Abate, A. How to Make over 20% Efficient Perovskite Solar Cells in Regular (n-i-p) and Inverted (p-i-n) Architectures. *Chemistry of Materials* **2018**, *30* (13), 4193–4201. https://doi.org/10.1021/acs.chemmater.8b00136.

(2) Giannozzi, P.; Baroni, S.; Bonini, N.; Calandra, M.; Car, R.; Cavazzoni, C.; Ceresoli, D.; Chiarotti, G. L.; Cococcioni, M.; Dabo, I.; Corso, A. D.; de Gironcoli, S.; Fabris, S.; Fratesi, G.; Gebauer, R.; Gerstmann, U.; Gougoussis, C.; Kokalj, A.; Lazzeri, M.; Martin-Samos, L.; Marzari, N.; Mauri, F.; Mazzarello, R.; Paolini, S.; Pasquarello, A.; Paulatto, L.; Sbraccia, C.; Scandolo, S.; Sclauzero, G.; Seitsonen, A. P.; Smogunov, A.; Umari, P.; Wentzcovitch, R. M. QUANTUM ESPRESSO: A Modular and Open-Source Software Project for Quantum of Materials. *Journal of Physics: Condensed Matter* **2009**, *21* (39), 395502. https://doi.org/10.1088/0953-8984/21/39/395502.

(3) Perdew, J. P.; Burke, K.; Ernzerhof, M. Generalized Gradient Approximation Made Simple. *Phys Rev Lett* **1996**, *77* (18).

(4) Grimme, S.; Antony, J.; Ehrlich, S.; Krieg, H. A Consistent and Accurate Ab Initio Parametrization of Density Functional Dispersion Correction (DFT-D) for the 94 Elements H-Pu. *Journal of Chemical Physics* **2010**, *132* (15). https://doi.org/10.1063/1.3382344.

(5) Walsh, A.; eldS22; Brivio, F.; Frost, J. M. WMD-Group/Hybrid-Perovskites: Collection 1. **2019**. https://doi.org/10.5281/ZENODO.2641358.

(6) Yin, J.; Naphade, R.; Gutiérrez Arzaluz, L.; Brédas, J. L.; Bakr, O. M.; Mohammed, O. F. Modulation of Broadband Emissions in Two-Dimensional â ¨100â ©-Oriented Ruddlesden-Popper Hybrid Perovskites. *ACS Energy Lett* **2020**, *5* (7), 2149–2155. https://doi.org/10.1021/acsenergylett.0c01047.

(7) T. Kellersohn; E. Alici; D. Esser; H.D. Lutz. Pb(IO3)2 I – Das Erste Halogenat Eines Zweiwertigen Hauptgruppenmetalls Mit Schichtenstruktur – Kristallstruktur, IR- Und Ramanspektren. *Zeitschrift fue Kristallographie- Crystalline Materials* **1993**, *203* (2), 225–233. https://doi.org/doi:10.1524/zkri.1993.203.Part-2.225.

(8) Frisch, M. J.; Trucks, G. W.; Schlegel, H. B.; Scuseria, G. E.; Robb, M. A.; Cheeseman, J. R.; Scalmani, G.; Barone, V.; Petersson, G. A.; Nakatsuji, H.; et al. Gaussian 09, Revision D. 01. *Gaussian, Inc.* Wallingford CT 2016.

(9) Cossi, M.; Rega, N.; Scalmani, G.; Barone, V. Energies, Structures, and Electronic Properties of Molecules in Solution with the C-PCM Solvation Model. *J Comput Chem* **2003**, *24* (6), 669–681. https://doi.org/10.1002/jcc.10189.

(10) Becke, A. D. Density-Functional Thermochemistry. III. The Role of Exact Exchange. *J Chem Phys* **1993**, *98* (7), 5648–5652. https://doi.org/10.1063/1.464913.

(11) Gil-González, E.; Perejón, A.; Sánchez-Jiménez, P. E.; Medina-Carrasco, S.; Kupčík, J.; Šubrt, J.; Criado, J. M.; Pérez-Maqueda, L. A. Crystallization Kinetics of Nanocrystalline Materials by Combined X-Ray Diffraction and Differential Scanning Calorimetry Experiments. *Cryst Growth Des* **2018**, *18* (5), 3107–3116. https://doi.org/10.1021/acs.cgd.8b00241.

(12) Sheridan, A. K.; Anwar, J. Kinetics of the Solid-State Phase Transformation of Form β to γ of Sulfanilamide Using Time-Resolved Energy-Dispersive x-Ray Diffraction. *Chemistry of Materials* **1996**, *8* (5), 1042–1050. https://doi.org/10.1021/cm950349z.

(13) Qin, M.; Xue, H.; Zhang, H.; Hu, H.; Liu, K.; Li, Y.; Qin, Z.; Ma, J.; Zhu, H.; Yan, K.; Fang, G.; Li, G.; Jeng, U. S.; Brocks, G.; Tao, S.; Lu, X. Precise Control of Perovskite Crystallization Kinetics via Sequential A-Site Doping. *Advanced Materials* **2020**, *32* (42). https://doi.org/10.1002/adma.202004630.

(14) Park, B.-W.; Kwon, H. W.; Lee, Y.; Lee, D. Y.; Kim, M. G.; Kim, G.; Kim, K. jeong; Kim, Y. K.; Im, J.; Shin, T. J.; Seok, S. Il. Stabilization of Formamidinium Lead Triiodide α-Phase with Isopropylammonium Chloride for Perovskite Solar Cells. *Nat Energy* **2021**, *6* (4), 419–428. https://doi.org/10.1038/s41560-021-00802-z.

(15) Lin, W. C.; Lo, W. C.; Li, J. X.; Wang, Y. K.; Tang, J. F.; Fong, Z. Y. In Situ XPS Investigation of the X-Ray-Triggered Decomposition of Perovskites in Ultrahigh Vacuum Condition. *Npj Mater Degrad* **2021**, *5* (1). https://doi.org/10.1038/s41529-021-00162-9.

(16) Moulder, J. F.; Stickle, W. F.; Sobol, P. E.; Bomber, K. D. *Handbook of X-Ray Photoelectron Spectroscopy*, 1995th ed.; Chastain, J., King, R. C., Eds.; Physical Electronics, Inc.: Eden Praire, Minnesota, 1992.

(17) Ciria-Ramos, I.; Navascués, N.; Diaw, F.; Furgeaud, C.; Arenal, R.; Ansón-Casaos, A.; Haro, M.; Juarez-Perez, E. J. Formamidinium Halide Salts as Precursors of Carbon Nitrides. *Carbon N Y* **2022**, *196*, 1035–1046. https://doi.org/10.1016/j.carbon.2022.05.051.

(18) Solanki, A.; Tavakoli, M. M.; Xu, Q.; Dintakurti, S. S. H.; Lim, S. S.; Bagui, A.; Hanna, J. V.; Kong, J.; Sum, T. C. Heavy Water Additive in Formamidinium: A Novel Approach to Enhance Perovskite Solar Cell Efficiency. *Advanced Materials* **2020**, *32* (23). https://doi.org/10.1002/adma.201907864.

(19) Hills-Kimball, K.; Nagaoka, Y.; Cao, C.; Chaykovsky, E.; Chen, O. Synthesis of Formamidinium Lead Halide Perovskite Nanocrystals through Solid-Liquid-Solid Cation Exchange. *J Mater Chem C Mater* **2017**, *5* (23), 5680–5684. https://doi.org/10.1039/c7tc00598a.

(20) Yu, X.; Qin, Y.; Peng, Q. Probe Decomposition of Methylammonium Lead Iodide Perovskite in N2 and O2 by in Situ Infrared Spectroscopy. *Journal of Physical Chemistry A* **2017**, *121* (6), 1169–1174. https://doi.org/10.1021/acs.jpca.6b12170.

(21) Siegler, T. D.; Dunlap-Shohl, W. A.; Meng, Y.; Yang, Y.; Kau, W. F.; Sunkari, P. P.; Tsai, C. E.; Armstrong, Z. J.; Chen, Y. C.; Beck, D. A. C.; Meilă, M.; Hillhouse, H. W. Water-Accelerated Photooxidation of CH3NH3PbI3 Perovskite. *J Am Chem Soc* **2022**, *144* (12), 5552–5561. https://doi.org/10.1021/jacs.2c00391.

(22) Wei, J.; Wang, Q.; Huo, J.; Gao, F.; Gan, Z.; Zhao, Q.; Li, H. Mechanisms and Suppression of Photoinduced Degradation in Perovskite Solar Cells. *Adv Energy Mater* **2021**, *11* (2002326), 1–31. https://doi.org/10.1002/aenm.202002326.

(23) Godding, J. S. W.; Ramadan, J.; Lin, Y.; Snaith, H. J.; Godding, J. S. W.; Ramadan, A. J.; Lin, Y.; Schutt, K.; Snaith, H. J. Oxidative Passivation of Metal Halide Perovskites. *Joule* **2019**, *3* (11), 2716–2731. https://doi.org/10.1016/j.joule.2019.08.006.

(24) Aristidou, N.; Eames, C.; Sanchez-molina, I.; Bu, X.; Kosco, J.; Islam, M. S.; Haque, S. A. Fast Oxygen Diffusion and Iodide Defects Mediate Oxygen-Induced Degradation of Perovskite Solar Cells. *Nat Commun* **2017**, *8* (May), 1–10. https://doi.org/10.1038/ncomms15218.

(25) Hayyan, M.; Hashim, M. A.; Alnashef, I. M. Superoxide Ion: Generation and Chemical Implications. *Chemical Reviews*. American Chemical Society March 9, 2016, pp 3029–3085. https://doi.org/10.1021/acs.chemrev.5b00407.

(26) Moot, T.; Dikova, D. R.; Hazarika, A.; Schloemer, T. H.; Habisreutinger, S. N.; Leick, N.; Dunfield, S. P.; Rosales, B. A.; Harvey, S. P.; Pfeilsticker, J. R.; Teeter, G.; Wheeler, L. M.; Larson, B. W.; Luther, J. M. Beyond Strain: Controlling the Surface Chemistry of CsPbI3Nanocrystal Films for Improved Stability against Ambient Reactive Oxygen Species. *Chemistry of Materials* **2020**, *32* (18), 7850–7860. https://doi.org/10.1021/acs.chemmater.0c02543.
